# ZDHHC9: a promising therapeutic target for triple-negative breast cancer through immune modulation and immune checkpoint blockade resistance

**DOI:** 10.1007/s12672-023-00790-4

**Published:** 2023-10-24

**Authors:** Chao Xu, Yongjie Xie, Peng Xie, Jianming Li, Zhongsheng Tong, Yanfang Yang

**Affiliations:** 1 Key Laboratory of Breast Cancer Prevention and Therapy, Tianjin Medical University, Ministry of Education, North Huanhu West Road, Tianjin, 300060 China; 2 National Clinical Research Center for Cancer, Tianjin Cancer Hospital Airport Hospital, Tianjin, 300060 China; 3 Key Laboratory of Cancer Prevention and Therapy, Tianjin Medical University Cancer Institute and Hospital, National Clinical Research Center for Cancer, Tianjin’s Clinical Research Center for Cancer, Tianjin, 300060 China

**Keywords:** TNBC, ZDHHC9, Immune biomarkers, Immunotherapy resistance

## Abstract

**Background:**

Triple-negative breast cancer (TNBC) is a subtype of breast cancer with limited treatment options and poor prognosis. This study aimed to identify potential therapeutic targets based on the expression profiles of differentially expressed genes (DEGs) in TNBC.

**Methods:**

The Limma package was used to identify DEGs in TCGA and GEO datasets. Immunohistochemical (IHC) analysis and western blotting were used to determine the expression of ZDHHC9 in TNBC tissues. Flow cytometry assay and tissue immunofluorescence analysis were used to detect infiltration of multiple immune cells in tumor tissue at different levels of ZDHHC9 expression.

**Results:**

ZDHHC9 was identified as a key factor associated with resistance to ICB therapy through weighted gene co-expression network analysis (WGCNA) and single-cell RNA sequencing (scRNA-seq). Subsequently, immunohistochemical (IHC) analysis and western blotting verified that ZDHHC9 expression was elevated in TNBC cancer tissues and that elevated expression of ZDHHC9 was associated with the poor survival of patients with TNBC. Analysis of data from several public datasets revealed that patients with high ZDHHC9 expression had an increased proportion of Ki-67 + breast cancer cells and tended to be basal-like breast cancer. In addition, in vitro and in vivo experiments demonstrated that high expression of ZDHHC9 significantly predicted the efficacy and responsiveness of immunotherapy in TNBC.

**Conclusion:**

These findings suggest that ZDHHC9 is a valuable marker for guiding the classification, diagnosis and prognosis of TNBC and developing specific targeted therapies.

**Supplementary Information:**

The online version contains supplementary material available at 10.1007/s12672-023-00790-4.

## Introduction

Breast cancer is the most common malignancy in women, accounting for almost one-third of all cancer cases among women, and is the second leading cause of cancer-related death in women [1]. Based on different molecular stages, breast cancer can be classified as luminal A, luminal B, human epidermal growth factor receptor 2 (HER2)-positive and triple-negative breast cancer (TNBC) [2]. TNBC, which accounts for approximately 15–20% of all breast cancer cases, lacks the expression of hormone (oestrogen and progesterone) receptors and HER2 and is characterised by high malignancy, high recurrence rates and a poor prognosis [3]. Owing to the lack of targeted therapies, chemotherapy remains the mainstay of treatment for advanced TNBC [4]. However, chemotherapy is not the ideal treatment option for advanced TNBC because it has highly toxic side effects and a short effective duration of action and patients may readily develop drug resistance.

Immunotherapy is a promising cancer treatment that has been developed at a rapid pace in recent years [5]. Immune checkpoint inhibitors, including monoclonal antibodies against PD-1, PD-L1 and CTLA-4, are one of the most successful classes of immunotherapeutic agents that produce durable anti-tumor immune responses by blocking immunosuppressive receptors [6]. Although positive therapeutic outcomes have been achieved in various solid tumors, including non-small cell lung cancer and melanoma [7–9], and haematological tumors [10], only some patients with TNBC have been shown to benefit from immune checkpoint blockade (ICB) therapy [11]. The reasons why most patients with TNBC do not respond to ICB therapy or fail to maintain a durable response warrant in-depth investigation.

ZDHHC9 is a member of the ZDHHC protein acyltransferase (ZDHHC) family that comprises 23 members, namely, ZDHHC1–ZDHHC9 and ZDHHC11–ZDHHC24, which mediate the palmitoylation of proteins [12]. Palmitoylation is a type of post-translational modification that involves reversible lipid modifications. It enhances the hydrophobicity of proteins, leading to changes in protein translocation, membrane localisation, protein stability, protein interactions and signal transduction, and critically affects cell proliferation and metastasis, chemoresistance, immunosuppression and angiogenesis in tumors [13]. Loss-of-function mutations in ZDHHC9 trigger X-linked intellectual disability (XLID) and are associated with an increased risk of epilepsy [14]. ZDHHC9-mediated GLUT1 S-palmitoylation promotes glycolysis and tumorigenesis in glioblastoma [15]. ZDHHC9-mediated palmitoylation maintains the stability and cell surface distribution of PD-L1, leading to immune escape of breast cancer cells [16], whereas overexpression of ZDHHC9 greatly inhibits the proliferation of colorectal cancer cells (21). However, the expression of ZDHHC9 in breast cancer subtypes, including TNBC, and its effects on immune cell infiltration within the tumor have not been reported.

In this study, bioinformatic analysis revealed four genes (TBC1D24, TRIM67, QRSL1 and ZDHHC9) that were highly expressed in TNBC and were associated with resistance to ICB therapy. Of the four genes, ZDHHC9 was identified as a key gene associated with resistance to ICB through weighted gene co-expression network analysis (WGCNA) and single-cell RNA sequencing (scRNA-seq). Immunohistochemical (IHC) analysis and western blotting validated that overexpression of ZDHHC9 in TNBC tissues was associated with a poor prognosis. Knockdown of ZDHHC9 in the murine TNBC cell line 4T1 improved the tumor immune microenvironment, which may partly explain the cause of resistance to ICB therapy and provide a theoretical basis for combination therapy.

## Materials and methods

### Data acquisition and pre-processing

The mRNA expression data and corresponding phenotypic data of 233 primary TNBC specimens were extracted from TCGA database and pre-processed for subsequent analysis. All the GSE48390, GSE58812, GSE20685, GSE162228, GSE42568, GSE20711, GSE88770, GSE61304, GSE21653, GSE97342 and GSE9893 datasets were downloaded from GEO database for analysis. The prediction of immunotherapy responsiveness and the validation of the multi-centre cohort in this study were mainly obtained from a local and web-based software tool called BEST, developed by a team from Zhengzhou University in China and some members of our team. The URL is as follows: https://rookieutopia.com/app_direct/BEST/. The gene expression data were subjected to log2 transformation (normalised RSEM count of 1), and genes with low or no expression were removed (genes with an average count of > 1 and expressed in > 75% of patients). For quality control, relative log expression (RLE) and standardised scale-free standard error (NUSE) were computed using the affyPLM package. The original gene expression data were background-corrected using the robust multi-array average (RMA) algorithm, standardised using a quantitative method and summarised using the topic polishing method. MSigDB was used to obtain genes related to cell biology. All patients with primary TNBC in TCGA and GEO datasets were selected to screen characteristic genes and establish a TNM staging prognostic/risk model. Bayes test was performed (FDR < 0.05) to determine differentially expressed genes (DEGs) among multiple samples in the GEO dataset.

### Screening of differentially expressed genes

The Limma package was used to identify DEGs in TCGA and GEO datasets, and the results of mRNA sequencing of DEGs were visualised on a volcano plot. A Venn diagram and volcano plot were constructed using ggplot2. Adjusted (Adj.) p-values of < 0.05 were considered statistically significant.

### Gene ontology and kyoto encyclopedia of genes and genomes pathway enrichment analyses

The cluster Profiler package was used to perform Gene Ontology (GO) and Kyoto Encyclopedia of Genes and Genomes (KEGG) enrichment analyses. Adj. p-values of < 0.05 were considered statistically significant. DEGs were subjected to KEGG/GO enrichment analyses, and the results were visualised on a histogram and bubble plot. The gene set enrichment analysis (GSEA) method was used to identify pathways associated with the expression of DEGs, and the groups were arranged 10,000 times in each analysis. Adj. p-values of < 0.05 and FDR values of < 0.25 indicated significant differences, and ‘c2.cp.kegg.v7.0.symbols.gmt’ was used as the reference gene set. The results of enrichment analysis were characterised based on adj. p-values and NESs.

### Prognostic markers screened via Lasso–Cox regression

The glmnet package was used to implement Lasso–Cox regression analysis to determine genes associated with the prognosis of TNBC. Genes identified as covariates in univariate analysis were included in multivariate Cox regression analysis to determine their impact on overall survival (OS). A prognostic model was established by integrating the expression levels of the selected genes and the multivariate Cox regression coefficients. The model was used to stratify patients with TNBC using the median risk score as the threshold. Patients with a risk score greater than the median risk score were assigned to the high-risk group, whereas the remaining patients were assigned to the low-risk group. The log-rank test was performed to evaluate differences in the survival rate between the two groups, and a calibration curve was plotted using the fitplot package.

### Estimation of the risk model

A time-dependent receiver operating characteristic (ROC) curve was plotted to evaluate the predictive accuracy of the risk model. The sensitivity and specificity of predicting the survival rate of patients with TNBC were calculated at 1-, 3- and 5-year follow-up intervals. Decision curve analysis (DCA) was used to assess the predictive accuracy of the risk model, and the results were corroborated using a calibration curve. The risk score and other clinical indicators, such as age, sex, TNM stage and histological grade, were also observed. The distribution of death events was demonstrated on a point map based on the increase in the risk score. A heatmap was constructed to demonstrate the distribution of the expression of each characteristic gene in the high- and low-risk groups. To assess the independent predictive performance and reliability of the prognostic model, common indicators such as age, TNM stage and histological grade were used to predict the prognosis of TNBC. Univariate and multivariate Cox regression analyses were performed based on the clinical characteristics and risk scores of patients with TNBC in TCGA cohort to determine clinical factors associated with survival. The log-rank test was performed to verify whether the risk score was related to other survival-related clinical characteristics. Subsequently, variables that were identified as independent prognostic factors were used to establish a nomogram. By intersecting the above prognostic genes in triple-negative breast cancer, we analyzed OS, RFS, PFS, DSS from GSE58812, GSE88770, GSE20685, GSE42568, GSE97342, GSE162228, GSE48390, GSE58812, GSE88770, GSE20711, GSE42568, GSE162228, GSE9893 datasets in GEO database.

### Assessment of immune infiltration

The CIBERSORT algorithm (URL: https://cibersort.stanford.edu/) was used to evaluate the infiltration of immune cells based on the RNA-Seq data of TNBC tissues. CIBERSORT provides gene expression signatures of 24 immune cell types, such as B cells, CD4+T cells, CD8+T cells, neutrophils, macrophages and dendritic cells.

### Analysis of tumor burden in triple-negative breast cancer

The cBioPortal database is a molecular dataset that helps to understand genetics, epigenetics, gene expression and proteomics for oncology and cytology research [17]. It provides an interactive interface for custom data that allows researchers to explore the relationship between genetic changes and clinical research. The mutation frequency and percentage of key prognostic genes in triple-negative breast cancer were analysed, and clinical characteristics, such as clinical stage, pathological grade and expression of tumor-specific markers, were shown through the public database (http://www.cbioportal.org/).

### Histochemical analysis of key prognostic genes

The Human Protein Atlas (HPA) database is based on proteomics, transcriptomics and systems biology data. It can be used to map tissues, cells and organs and contains the protein expression data of not only tumor tissues but also normal tissues. In addition, it provides survival curves for patients with tumors. In this study, the HPA database was used to evaluate the expression of the identified prognostic genes in TNBC based on histological staining.

### WGCNA

The gene expression data of patients with TNBC were extracted from TCGA. The median absolute deviation (MAD) of each gene was calculated, and the top 50% of genes with the smallest MAD were removed. Subsequently, the good Samples Genes tool in the R package WGCNA was used to eliminate outliers and construct a scale-free co-expression network. To construct the network, all pairwise genes were analysed via Pearson correlation analysis and the average linkage method. Subsequently, a weighted adjacency matrix was constructed using a power function: A_mn =|C_mn|^β (C_mn = Pearson correlation coefficient between Gene_m and Gene_n; A_mn = adjacency between Gene m and Gene n). In this equation, β represents a soft-thresholding parameter that emphasises strong correlations between genes and penalises weak correlations. After selecting the power of 7, the adjacency matrix was transformed into a topological overlap matrix (TOM) that measured the network connectivity of a gene defined as the sum of its adjacency with all other genes for generating a network, and the corresponding dissimilarity (1-TOM) was calculated. To classify genes with similar expression profiles into gene modules, average linkage hierarchical clustering was performed according to TOM-based dissimilarity, with a minimum number (gene group) set to 30 genes for constructing a dendrogram. For further analysis of these modules, the dissimilarity of module eigengenes was calculated, a cut-off line was selected for the module dendrogram and some modules were merged. Additionally, modules with distances less than 0.25 were merged, and 9 co-expression modules were eventually obtained. Notably, a gene set that could not be assigned to any module was included in the grey module. To screen for hub genes, the correlation between the module feature vector and gene expression was evaluated to obtain MM. Highly correlated genes within a clinically significant module were identified as hub genes based on the cut-off criteria (|MM|> 0.8).

### Cell culture and transfection

The murine TNBC cell line 4T1 and the human TNBC cell line MDA-MB-231 were obtained from the Type Culture Collection Committee of the Chinese Academy of Sciences (Shanghai, China). The cell lines were authenticated through short tandem repeat analysis. Mycoplasma contamination was not observed in the cell lines. 4T1 and MDA-MB-231 cells were cultured in RPMI-1640 medium and Dulbecco’s modified Eagle’s medium (DMEM) supplemented with 10% foetal bovine serum (FBS), respectively. The cultures were maintained in a humidified atmosphere of 95% air and 5% CO_2_ at 37 °C.

The shRNA sequences of the ZDHHC9 gene were synthesised and cloned into the pLV-H1-EF1α-puro vector. The lentivirus was generated in 293 T cells and transfected into 4T1 cells. Of the two stable cell lines, the most efficient cell line was used for subsequent experiments. shRNA sequences are listed in Supplementary Table 2.

### Patients and tissue samples

A total of 114 TNBC tissues were retrospectively collected from patients with a histological diagnosis of TNBC who underwent modified radical mastectomy between January 2010 and January 2012 at the Tianjin Medical University Cancer Institute and Hospital, China. Data regarding the clinicopathological features of patients were collected retrospectively. In addition, eight pairs of fresh TNBC and para-cancerous tissues were collected during surgery between June and December 2022. Total protein was extracted from the tissues for detecting ZDHHC9 expression via western blotting. The use of these samples and patient information was approved by the Tianjin Medical University Cancer Institute and the ethics committee of the hospital in accordance with the ethical standards as laid down in the 1964 Declaration of Helsinki and its later amendments.

### Immunohistochemical analysis

After de-paraffinisation and hydration, TNBC tissue sections were subjected to antigen retrieval under alkaline conditions (EDTA, pH 9.0) and incubated with a primary antibody against ZDHHC9 (Proteintech, 24046-1-AP, 1:200) overnight at 4 ℃. The following day, the sections were treated with HRP-conjugated secondary antibodies at 37 ℃ for 1 h, and the bound secondary antibodies were stained with DAB (ZSGB-BIO, ZLI-9018). The final staining scores were calculated by multiplying the score for staining intensity by the score for staining range. Staining intensity was scored as follows: 0, negative; 1, low; 2, medium; 3, high. Staining range was scored as follows: 0, 0% staining; 1, 1–25% staining; 2, 26–50% staining; 3, 51–100% staining. The final score ranged from 0 to 9: < 2, negative (−); 2–3, low staining ( +); 4–6, moderate staining (+ +); > 6, high staining (+ +  + +). Five areas were randomly examined using a light microscope (100 × magnification). The samples were independently analysed by two pathologists who were informed of the clinical features and examination findings of the patients.

### Cellular immunofluorescence analysis

Cells (MDA-MB-231 and 4T1) were fixed with 4% fresh paraformaldehyde, permeabilised with 0.2% Triton X-100 and incubated with 5% goat serum for 1 h to block non-specific interactions. Subsequently, the cells were incubated with primary antibodies against ZDHHC9 (Proteintech, 24046-1-AP, 1:100) and F-actin (Abcam, ab130935, 1:200) overnight at 4 ℃. The following day, the cells were incubated with fluorescently labelled secondary antibodies (1:200) (Thermo Fisher Scientific, USA) for 1 h, mounted with a DAPI-containing antifade mounting medium (Vector Laboratories) and sealed with clear nail polish. The cells were extensively washed with PBS after each step. Finally, images were captured using a confocal fluorescence microscope (ZEISS, Germany).

### Tissue immunofluorescence analysis

Two sets of 114 PDAC tissues were used for immunological assessment of ZDHHC9 and CD8/NCR1 expression. The tissues were stained using the Opal 7-Color Manual IHC Kit according to the manufacturer’s instructions. Briefly, after de-paraffinisation and hydration, TNBC tissue sections were fixed via microwave irradiation for 20 min under appropriate repair conditions and incubated with primary antibodies diluted in a blocking solution overnight at 4 °C (Supplementary Table 3). The following day, the tissues were incubated with HRP-conjugated secondary antibodies for 10 min at room temperature and stained with a fluorescent dye for 10 min at room temperature. The steps of microwave-assisted fixation, primary and secondary antibody incubation and cross-linking of fluorescent signals were repeated until the sections were stained for all indicators. The nuclei were stained with DAPI, and the tissue slices were sealed. The samples were washed with TBST after each step. Finally, images were captured using a fluorescent microscope (KEYENEC, Japanese).

Tissue immunofluorescence (IF) values were independently determined by two pathologists who were under a duty of confidentiality regarding the clinical features, examination findings and IHC analysis results of patients. Five randomly selected areas were evaluated microscopically (200 × magnification). CD8 + and NCR1 + cells in the tumor stroma were used to visualise CD8 + T cells, and NK cells, respectively. Based on the results of IHC analysis, patients were divided into the high- and low-ZDHHC9-expression groups.

### Western blotting

Whole-cell extracts were prepared by lysing cells in RIPA buffer supplemented with a protease inhibitor cocktail (Sigma-Aldrich, USA). The extracted protein was quantified using a BCA protein assay kit (Thermo Fisher Scientific, USA). A total of 20 μg of extracted protein was separated via SDS-PAGE, transferred onto polyvinylidene fluoride (PVDF) membranes and incubated with 5% skim milk in TBST for 1 h at room temperature to block non-specific interactions. Subsequently, the membranes were incubated with primary antibodies against ZDHHC9 (1:1000; Proteintech, 24046-1-AP), GAPDH (1:5000; Proteintech, 60004-1-Ig) and β-actin (1:5000; Santa Cruz Biotechnology, SC-47778) overnight at 4 ℃. The following day, the membranes were incubated with secondary antibodies (goat anti-rabbit or mouse antibodies, 1:5000; Abmart), and protein bands were visualised using an ECL system (Millipore, USA).

### RT-PCR

Total mRNA was extracted using the TRIzol reagent. The quality and quantity of the extracted RNA were measured on an ND-1000 spectrophotometer (Nanodrop Technologies). cDNA was synthesised using the qScript cDNA synthesis kit (Quanta Biosciences) according to the manufacturer’s instructions. Quantitative PCR was performed using the SYBR Green Master Mix, and the amplified products were detected via agarose gel electrophoresis. β-actin was used as a loading control. The primers used for PCR are listed in Supplementary Table 2.

### Cell viability assay

Cell viability was assessed using the 3-(4,5-Dimethylthiazol-2-yl)-2,5-Diphenyltetrazolium Bromide (MTT) assay, following the described methods [18].

### Wound-healing and transwell assays

Wound healing and migration assays were conducted using the specified 4T1 cells, following an established protocol as previously described [19]. For the Transwell assay, a sixfold diluted Matrigel was added to the upper chamber of the Boyden cell to evaluate invasion. The cells that migrated to the bottom of the filter were stained using a three-step staining kit from Thermo Fisher Scientific. Subsequently, a microscope was utilized to count the migrated cells, and statistical analysis was performed.

### Animals and tumor model

Immunologically sound 4–6-week old female C57BL/6 mice were used for in vivo analysis. All mice were housed under specific pathogen-free conditions. All experimental procedures involving animals were approved by the Ethics Committee of the Tianjin Medical University and Hospital Cancer Institute and were performed in accordance with the policies and procedures recommended by the NIH Guide for the Care and Use of Laboratory Animals.

A total of 1 × 10^6^ tumor cells (4T1-scramble and 4T1-shZDHHC9) in phosphate-buffered saline (PBS) were subcutaneously injected into the lateral abdomen of C57BL/6 mice. The width and length of tumors were measured every 4 days using an electronic calliper, and tumor volume was calculated using the following formula: volume = length × width^2^/2. The tumor volumes of the mice in this experiment did not exceed the maximum size or burden required by the ethics committee. The maximum diameter of tumours in mice was set at 15 mm, and the maximum volume was limited to 1500 mm^3^.

### Flow cytometry

Tumors were treated with 1 mg/mL collagenase, 2.5 U/mL hyaluronidase and 0.1 mg/mL DNase to produce single-cell suspensions. The abundance of tumor-infiltrating CD8 + T cells (CD3 + and CD8 +), CD4 + T cells (CD3 + and CD4 +), Treg cells (CD4 + , CD25 + and FOXP3 +), NK cells (NK1.1 +) and exhausted CD8 + T cells (PD-1 + and Tim3 +) was evaluated. Isotypic controls were used as negative controls. Data were analysed using the FCS Express 7 software. Relevant antibodies are listed in Supplementary Table 3.

### Statistical analysis

The R (version 3.4.0.3) and SPSS Statistics (version 22) software were used for statistical analysis. The Coxph function was used to implement univariate and multivariate Cox regression analyses, and the glmnet package in R was used to perform Lasso–Cox regression analysis. Additionally, the log-rank test was performed using the survdiff function in the survival package, and time-dependent ROC analysis was performed using the timeROC package. The characteristic gene expression profiles were demonstrated on heatmaps created using the ggplot2 package. The rms package in R was used to establish and use a nomogram. Pathway enrichment analysis was performed using the clusterProfiler package in R. Each experiment was performed independently at least three times. Data were expressed as the mean ± squared difference (SD). Student’s t-test was used to compare the means of two groups. The median survival time was estimated using the Kaplan–Meier method, and differences in survival between groups were examined using the log-rank test. Spearman rank correlation analysis was used to examine the correlation between parameters. Two-way analysis of variance and post hoc analysis with repeated measures (time × tumor volume) were performed to test for differences in tumor growth in mice among different groups. Statistical tests were performed using the GraphPad Prism 8 software. All statistical tests were two-tailed, and p-values of < 0.05 were considered statistically significant. Significant values were marked with an asterisk and indicated as follows: ns, p ≥ 0.05; *p < 0.05; **p < 0.01; ***p < 0.001; ****p < 0.0001.

## Results

### Markers associated with the prognosis and immune microenvironment of triple-negative breast cancer

**Fig. 1:**
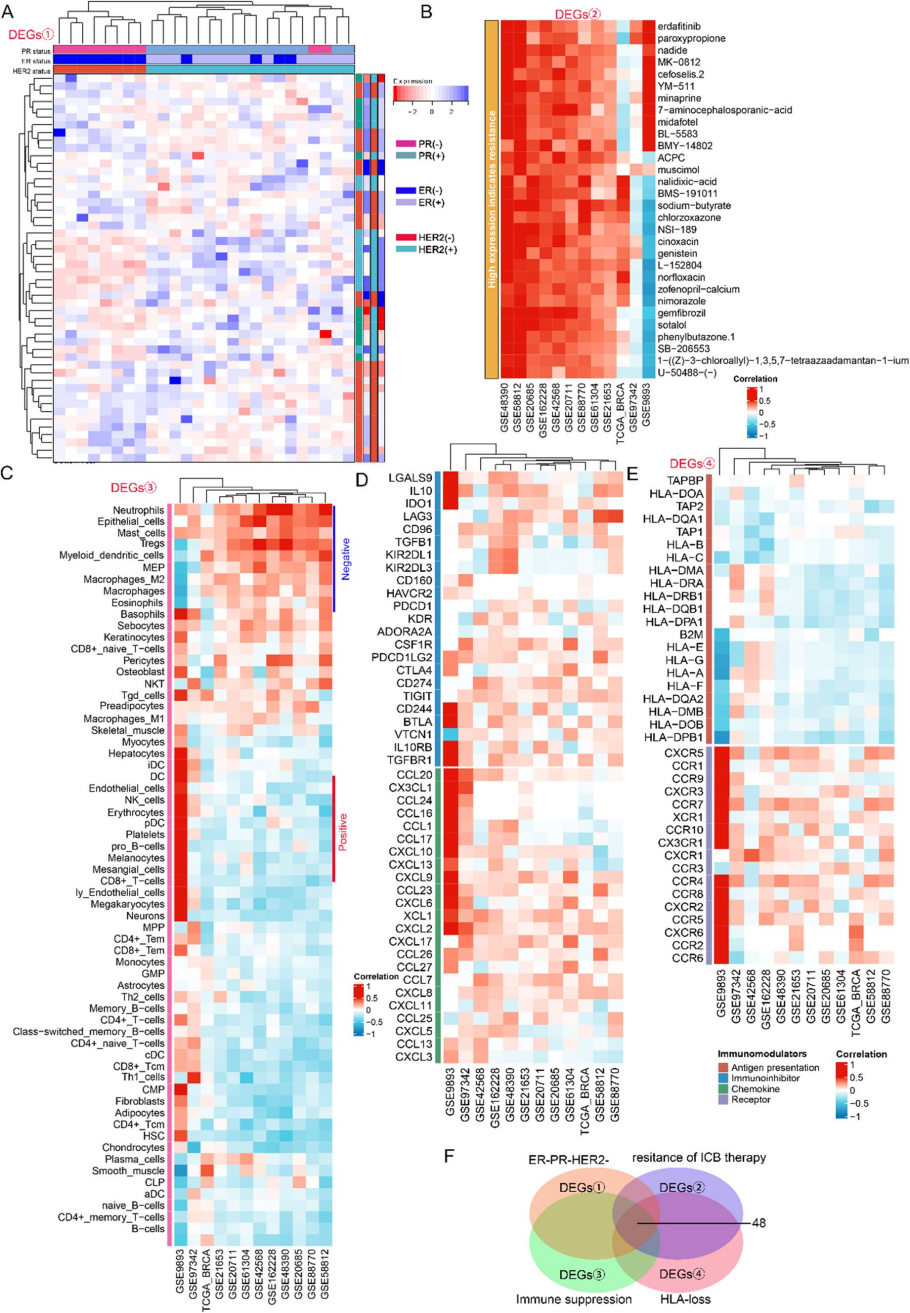
Markers associated with the prognosis and immune microenvironment of triple-negative breast cancer (TNBC). **A** Multi-dimensional heatmap demonstrating differentially expressed genes in all patients grouped based on the expression of oestrogen receptor (ER), progesterone receptor (PR) and human epidermal growth factor receptor 2 (HER2). The upregulated differential genes in patients with ER-negative, PR-negative and HER-2-negative breast cancer are shown in the volcano map. Intersecting genes among the three groups of patients were obtained. **B**–**E** Spearman correlation analysis of gene expression profiles that were positively correlated with resistance to immune checkpoint inhibitor (ICI) treatment, infiltration of immunosuppressive cells and expression of immunosuppressive checkpoints and negatively correlated with the expression of antigen-presenting molecules and immunosuppressive chemokines and receptors based on data from the GEO and TCGA databases. **F** Venn diagram demonstrating 48 differentially co-expressed genes identified by intersecting key genes from each expression profile with highly expressed genes in TNBC

In this study, we initially screened for genes associated with the prognosis and immune microenvironment of TNBC. The transcriptomic sequencing and clinicopathological data of patients with TNBC were extracted from TCGA database, which includes 233 TNBC specimens. Patients were divided based on the expression of ER, PR and HER-2, and DEGs among these patients were visualised on a multi-dimensional heatmap. A total of X, Y and Z upregulated genes were identified in patients with ER-negative, PR-negative and HER-2-negative breast cancer, respectively. These genes were intersected to obtain overlapping genes (Fig. 1A). Subsequently, Spearman correlation analysis was performed to identify gene expression profiles that were positively correlated with resistance to ICB treatment, infiltration of immunosuppressive cells and expression of immunosuppressive checkpoints and negatively correlated with the expression of antigen-presenting molecules and immunosuppressive chemokines and receptors in the GEO and TCGA datasets (Fig. 1B–E). Based on the abovementioned factors, key genes were extracted from each expression profile and intersected with genes highly expressed in TNBC. Eventually, 48 differentially co-expressed genes were identified and demonstrated on a Venn diagram (Fig. 1F). These genes are enlisted in Supplementary Table 1. The functional characteristics of these 48 genes were examined via KEGG and GO pathway enrichment analyses. The results revealed that these genes were mainly involved in the NF-κB signalling pathway, ECM–receptor pathway, negative regulation of T-cell-mediated cytotoxicity, Wnt signalling pathway and MAPK signalling pathway (Supplementary Fig. 1A, B). These gene markers may be used to guide the development of individualised treatment strategies for patients with TNBC.

### Identification and validation of four genes associated with the prognosis of patients with TNBC

**Fig. 2:**
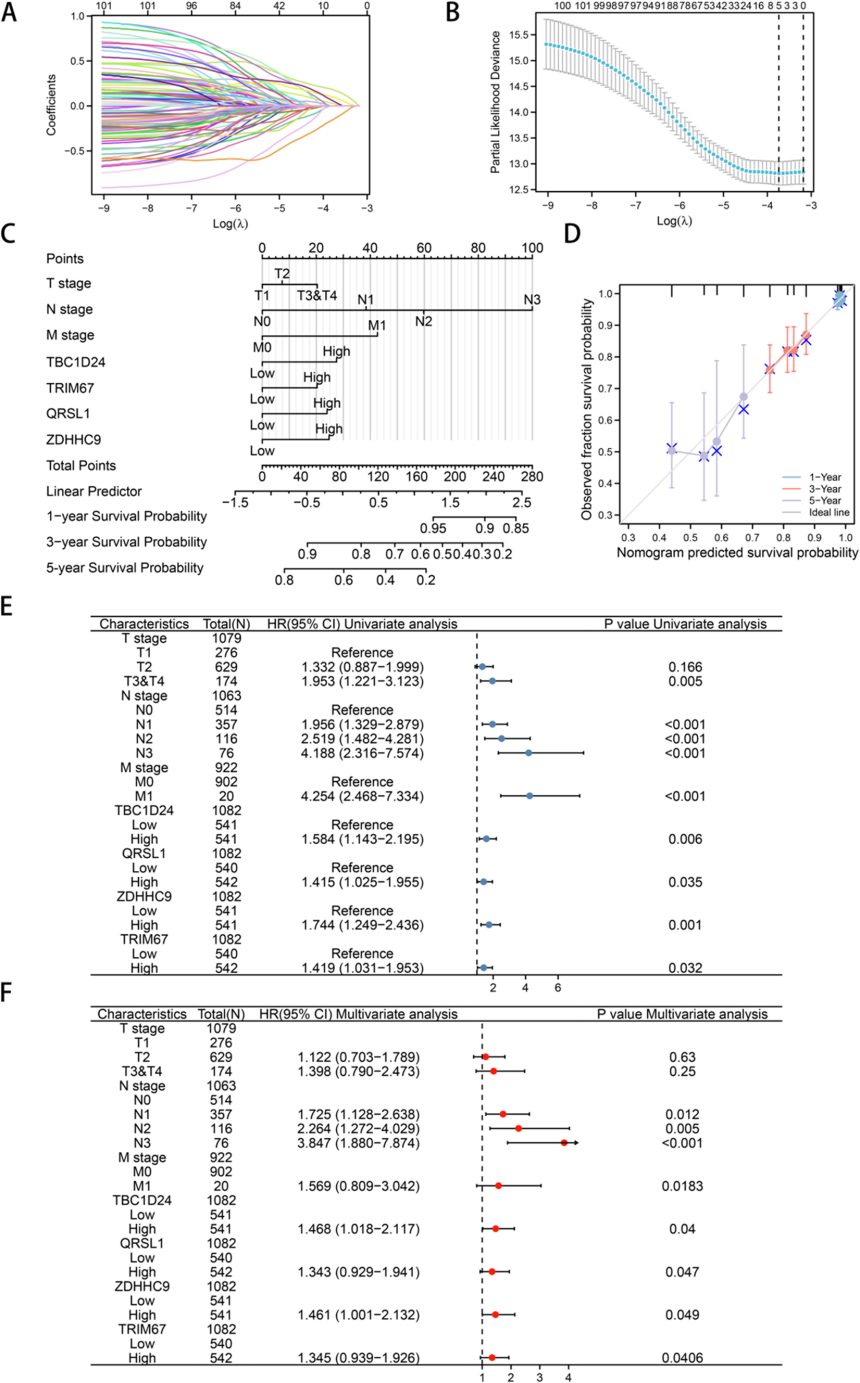
Identification and validation of four genes associated with the prognosis of patients with TNBC. **A**–**B** Lasso regression and matching λ values were used to select four genes: TBC1D24, YWHAB, ZDHHC9 and QRSL1. **C** A nomogram model integrating these four genes and clinical characteristics was constructed to predict the 1-, 3- and 5-year survival rates of patients with TNBC. **D** Calibration curve for validating the predictive efficiency of the model. **E**,** F** Univariate and multivariate Cox regression analyses of the four genes and clinical parameters. The hazard ratio (HR) of all genes was > 1, indicating that the selected genes significantly promoted the development of TNBC

Figure 1 demonstrates genes that are upregulated in TNBC. The sequencing data of patients with TNBC in TCGA cohort were used to screen for characteristic genes affecting the progression and prognosis of TNBC. Lasso regression and matching λ values were used to select four genes that significantly contributed to the prognosis of TNBC: TBC1D24, TRIM67, ZDHHC9 and QRSL1 (Fig. 2A, B). Subsequently, a nomogram model was constructed based on the combination of these four genes and clinical characteristics such as TNM stage (Fig. 2C) to predict the 1-, 3- and 5-year survival rates of patients with TNBC (Fig. 2D). All four genes had good predictive efficiency. Calibration curves were plotted to validate the efficiency of the model, and univariate and multivariate Cox regression analyses were performed to analyse the four genes and clinical parameters. The hazard ratios (HRs) of TBC1D24, ZDHHC9, TRIM67 and QRSL1 were 1.468 (p = 0.04), 1.461 (p = 0.049), 1.345 (p = 0.0406) and 1.343 (p = 0.047), indicating that these genes were significantly independent risk factors in the development of TNBC (Fig. 2E, F). In addition, ROC analysis revealed that the four genes had higher AUC values for predicting the diagnosis of breast cancer (TBC1D24, AUC = 0.798; TRIM67, AUC = 0.711; ZDHHC9, AUC = 0.810; QRSL1, AUC = 0.801) (Supplementary Fig. 2A–D). Further analysis revealed that the four genes were highly expressed in ER-negative, PR-negative and HER-2-negative breast cancer (Supplementary Fig. 2E–P). K-M analysis was performed to verify the correlation between clinical parameters and the expression of the four genes in TNBC. The results revealed that patients with high expression of the four genes had shorter OS (Supplementary Fig. 3A–D).

### Risk model for predicting the prognosis of triple-negative breast cancer

**Fig. 3:**
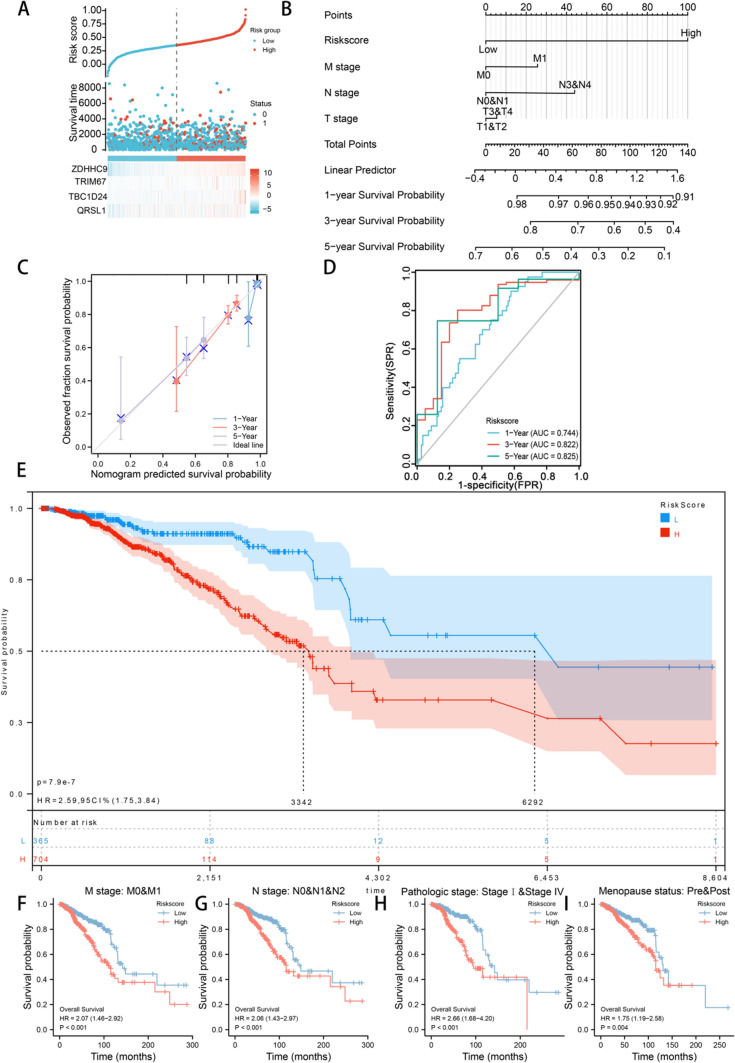
Risk model for predicting the prognosis of patients with triple-negative breast cancer. **A** The weighted risk score was identified as a significant predictor of survival and malignant progression in TNBC. Patients with high risk scores had poorer survival, which was associated with the high expression of the four genes. **B** A nomogram model incorporating the risk score and clinical parameters was constructed to predict the 1-, 3- and 5-year survival rates of patients with TNBC; the predictive efficiency of the risk score was compared with that of the traditional TNM staging classification. **C** Calibration curve for verifying the validity and consistency of the risk model. **D** Time-dependent ROC curves for predicting 1-, 3- and 5-year survival probabilities to validate the predictive efficiency of the model. **E** The median survival time was calculated in patients with TNBC with high risk scores in the GEO dataset. **F**−**I** Prediction of overall survival in patient subgroups categorised based on the primary therapeutic outcome, residual tumor, histological grade and N stage

To predict the cancer-promoting characteristics and prognosis of TNBC using the combination of the four genes, we calculated risk coefficients for co-expression integration and constructed a risk model with the following formula: risk score = 2*TBC1D24 + 1*TRIM67 + 1*QRSL1 + 1.8*ZDHHC9. The risk score was identified as a significant predictor of survival and malignant progression in patients with TNBC. In particular, patients with high risk scores had significantly shorter survival, which was associated with the high expression of the four genes (Fig. 3A). A nomogram constructed based on the risk score and clinical parameters indicated that the accuracy of the risk model for predicting 1-, 3- and 5-year survival probabilities was higher than that of the traditional TNM stage classification (Fig. 3B). In addition, the calibration curve demonstrated the validity and consistency of the model (Fig. 3C), and ROC curves validated the excellent predictive efficiency of the model for 1-year (AUC = 0.744), 3-year (AUC = 0.822) and 5-year (AUC = 0.825) survival probabilities (Fig. 3D).

To further validate the risk model, the sequencing data of patients with breast cancer were extracted from the GEO database. Patients with high risk scores had significantly shorter median survival (HR = 2.59, P = 7.9e-7) (Fig. 3E). In addition, the risk score could significantly predict OS in patient subgroups categorised based on the primary therapeutic outcome, residual tumor, histological grade and N stage (Fig. 3F–I).

### Weighted gene co-expression network analysis of TNBC samples with clinical features

**Fig. 4:**
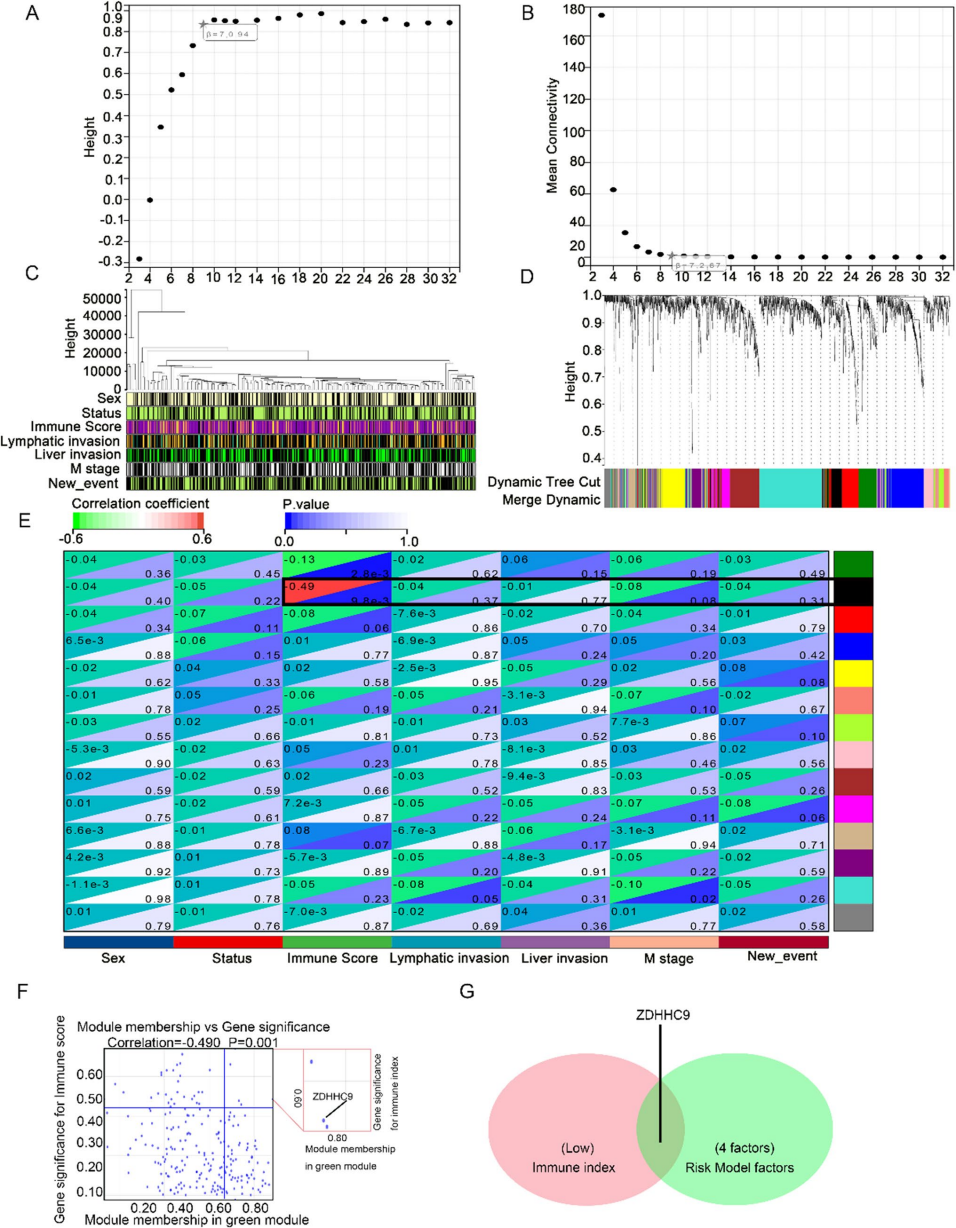
Weighted gene co-expression network analysis (WGCNA) of triple-negative breast cancer samples with clinical features. **A** Analysis of the scale-free fit index for various soft-thresholding powers (β). **B** Analysis of the mean connectivity for various soft-thresholding powers (β). **C** Sample dendrogram and trait heatmap. **D** Dendrogram of gene modules identified using the Dynamic Tree Cut algorithm. **E** Scatter plot of the correlation between the pink module and immune index. **F** Scatter plot of gene significance (GS) versus module membership (MM) in patients with TNBC. **G** Venn diagram demonstrating the intersection of target genes in the risk model and those in the weighted co-expression network. The analysis included 233 samples with clinical features. TOM was mapped to 1000 genes selected in the analysis, indicating that each module was independently validated

WGCNA is a systematic approach for effective identification of the expression patterns of multiple genes in samples with different clinical features and for generating gene sets with similar expression patterns. It enables exploration of the relationship between gene modules and sample phenotypes, such as clinical features [20]. In this study, a soft threshold of β value of 7 (scale-free R^2^ = 0.94) was selected to establish a scale-free network (Fig. 4A, B). A total of 233 samples with clinical data were examined via WGCNA (Fig. 4C). After removing the greyscale module using the Dynamic Tree Cut method, three co-expression modules were identified (Fig. 4D). TOM was mapped to 1000 genes selected in the analysis, indicating that each module was independently validated. The pink module, which contained ZDHHC9, was associated with the immune index (R = − 0.490, P = 0.001) (Fig. 4E). A scatter plot constructed between gene significance (GS) and module membership (MM) (color module and phenotype) (Fig. 4F) revealed ZDHHC9 as a key gene in the identified modules. As shown in the Venn diagram in Fig. 4G, ZDHHC9 was identified as a common target gene between the risk model and the weighted co-expression network (Fig. 4G).

### Investigation of the role of ZDHHC9 in TNBC using single-cell transcriptomic data

**Fig. 5:**
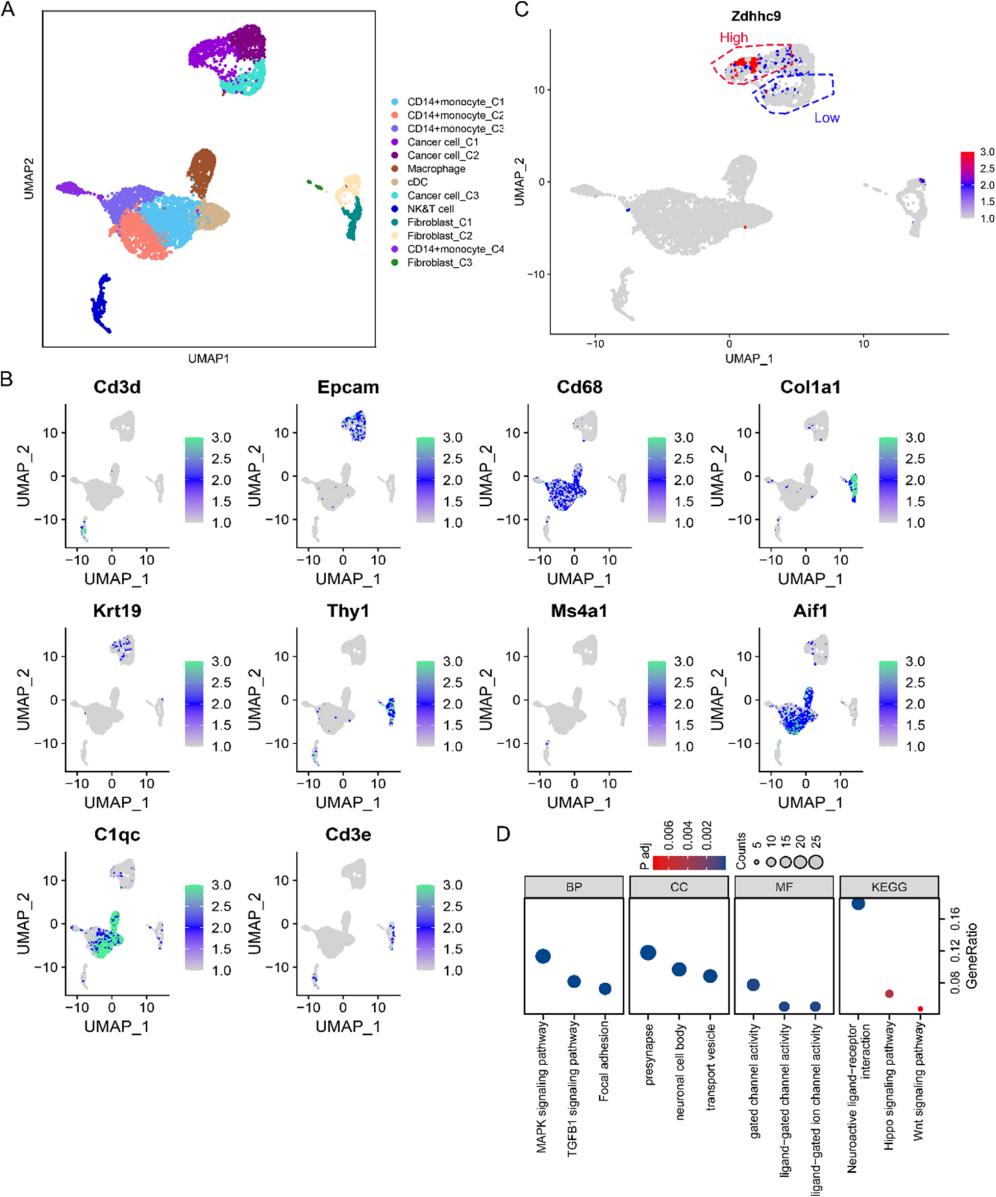
Investigation of the role of ZDHHC9 in triple-negative breast cancer using single-cell transcriptomic data. **A**,** B** Validation of epithelial cell type as malignant using EPCAM. **C** Identification of cell populations with high and low expression of ZDHHC9 via single-cell subgroup analysis. **D** KEGG pathway enrichment analysis of differentially expressed genes between cell populations with high and low expression of ZDHHC9. The analysis used single-cell transcriptomic data from the GSE12345 dataset

To investigate the role of ZDHHC9 in TNBC, the single-cell transcriptomic data of TNBC samples were extracted from the Dryad data repository (https://datadryad.org/stash/dataset/doi:10.6071/M3238R). Through EPCAM, we confirmed that the epithelial cell type was a malignant epithelium in the previous analysis (Fig. 5A, B). The malignant epithelial cell population was isolated via single-cell subgroup analysis, and cell populations with high and low expression of ZDHHC9 were identified (Fig. 5C). Subsequently, DEGs were identified between the two cell populations, and their functions were examined via KEGG pathway enrichment analysis. The genes were found to be associated with critical pathways such as TGFB1, Hippo and MAPK signalling pathways and focal adhesion-related pathways, which are involved in the development of breast cancer (Fig. 5D).

### ZDHHC9 potentially serves as an important marker for guiding the classification, diagnosis and prognosis of triple-negative breast cancer

**Fig. 6:**
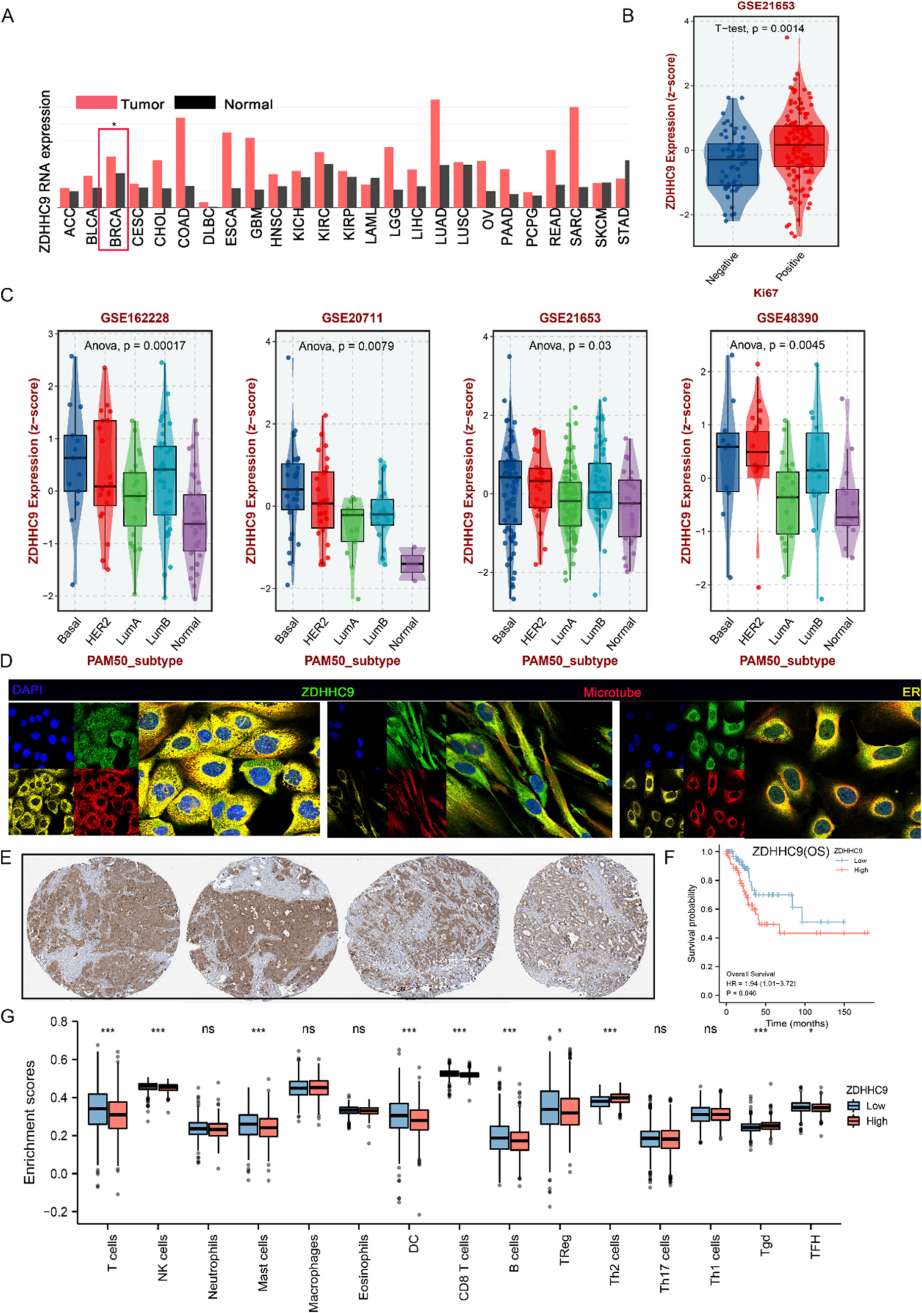
ZDHHC9 potentially serves as an important marker for guiding the classification, diagnosis and prognosis of triple-negative breast cancer **A** Heatmap demonstrating the expression of ZDHHC9 in different cancer types and their adjacent tissues based on TCGA database. The colour scale on the right indicates the expression level of ZDHHC9. **B** Box plot demonstrating the relationship between ZDHHC9 expression and Ki-67 + breast cancer cells. The Y-axis represents the expression level of ZDHHC9, and the X-axis represents the Ki-67 + expression status. **C** Forest plots demonstrating the relationship between ZDHHC9 expression and basal-like breast cancer in four independent datasets (GSE16228, GSE20711, GSE21653 and GSE48390). **D** Immunofluorescence images from the Human Protein Atlas (HPA) database demonstrating the subcellular localisation of ZDHHC9 in A-431, ASC52telo and U2OS cell lines. **E** Immunohistochemical images of ZDHHC9 in breast cancer tissues and adjacent tissues from the HPA database. **F** Kaplan–Meier survival analysis of patients with breast cancer in TCGA cohort according to ZDHHC9 expression. The survival curves are shown in red and green for high and low expression of ZDHHC9, respectively. **G** Bar plot demonstrating the relationship between ZDHHC9 expression and the infiltration of multiple immune cells such as CD8 + T cells, Treg cells and NK cells in breast cancer. The Y-axis represents the log10-transformed p-values of each immune cell type, and the X-axis represents the expression of ZDHHC9 (*p < 0.05; **p < 0.01; ***p < 0.001)

The expression of ZDHHC9 was examined at the pan-cancer level. It was higher in most cancer tissues, including breast cancer tissues, than in adjacent normal tissues (Fig. 6A). In addition, patients with high ZDHHC9 expression had an increased proportion of Ki-67 + breast cancer cells (Fig. 6B) and tended to have basal-like breast cancer in several datasets, including GSE16228, GSE20711, GSE21653 and GSE48390 datasets (Fig. 6C). Data extracted from HPA revealed that ZDHHC9 was primarily localised in the cytoplasm in A-431, ASC52telo and U2OS cells (Fig. 6D). In addition, IHC analysis revealed that ZDHHC9 expression was significantly higher in cancer tissues than in adjacent normal tissues (Fig. 6E). In the 233 breast cancer cases in TCGA cohort, ZDHHC9 expression significantly predicted a poor prognosis (HR = 1.94, p = 0.046) (Fig. 6F) and was significantly negatively correlated with the infiltration of CD8 + T cells and NK cells (p < 0.001) (Fig. 6G). These results suggest that high expression of ZDHHC9 is associated with an immunosuppressive microenvironment and that ZDHHC9 can serve as an important marker for guiding the classification, diagnosis and prognosis of breast cancer, including TNBC, and developing specific targeted therapies.

### High expression of ZDHHC9 correlates with a poor response to immunotherapy, activation of specific signalling pathways and poor survival outcomes in breast cancer

**Fig. 7:**
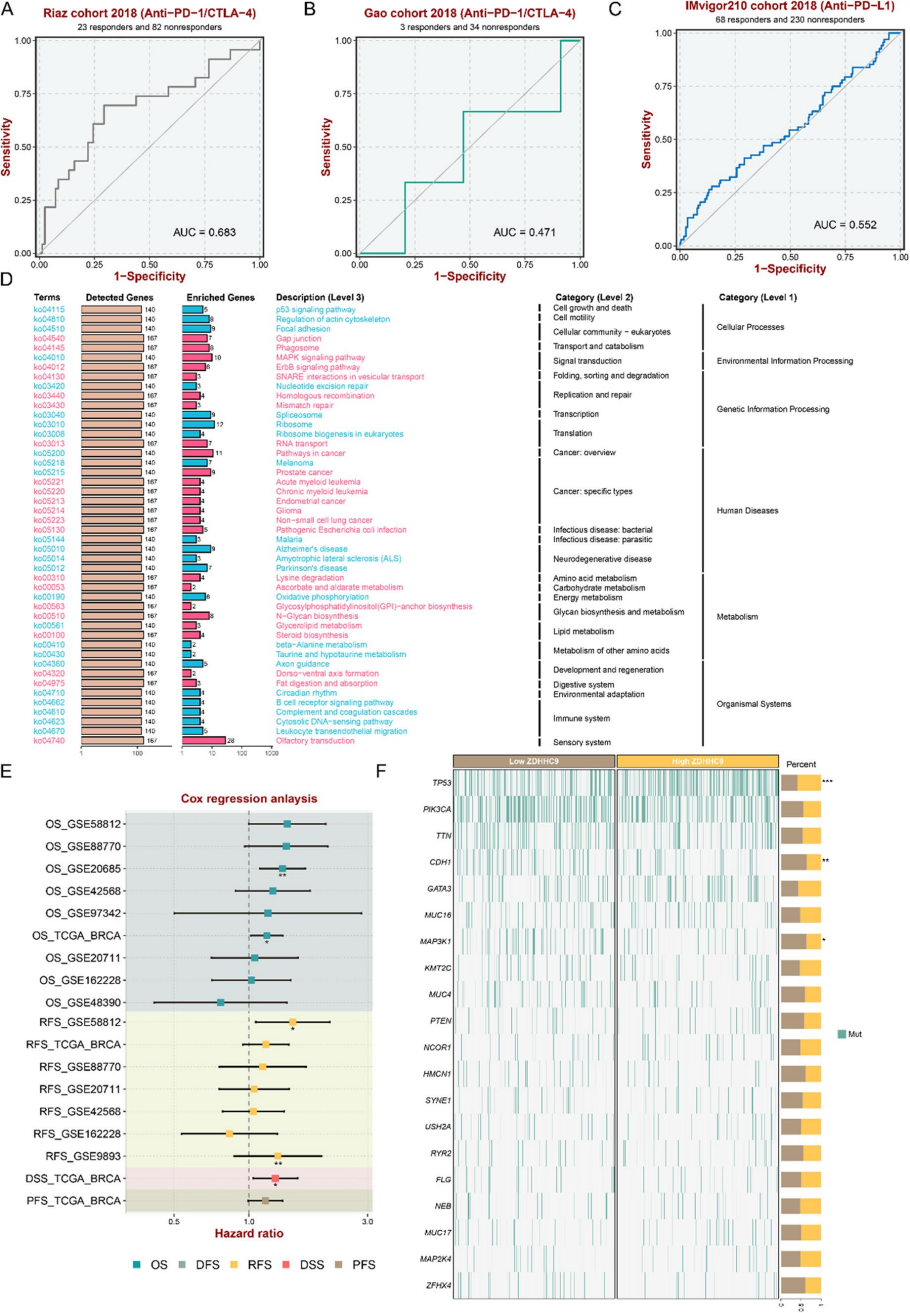
High expression of ZDHHC9 correlates with a poor response to immunotherapy, activation of specific signalling pathways and poor survival outcomes in breast cancer patients. **A**–**C** ROC curves demonstrating the efficacy and responsiveness of immunotherapy in patients with TNBC with high expression of ZDHHC9 in the Riaz 2018, Gao 2018 and IMvigor210 2018 cohorts. **D** Pathway enrichment analysis of ZDHHC9 in TNBC. High ZDHHC9 expression was positively correlated with the MAPK and ErbB signalling pathways and glycolysis-related pathway and negatively correlated with the P53 signalling pathway. **E** Kaplan–Meier survival curves of overall survival (OS), relapse-free survival (RFS), disease-specific survival (DSS) and progression-free survival (PFS) in patients with breast cancer with high expression of ZDHHC9 in a multi-centre retrospective cohort. **F** Mutations co-occurring with high expression of ZDHHC9 in a multi-centre cohort of patients with breast cancer. The most frequent mutation was TP53, followed by mutations in CDH1 and MAP3K1

Three cohort studies have revealed that the high expression of ZDHHC9 significantly predicts the efficacy and responsiveness of immunotherapy in TNBC. In this study, patients with TNBC with high ZDHHC9 expression were found to be less responsive to ICB therapy, with an average treatment response rate of < 20% than that in the abovementioned three studies (Riaz, 2018; Gao, 2018; IMvigor210 study, 2018) (Fig. 7A–C). Pathway enrichment analysis revealed that high ZDHHC9 expression in TNBC was mainly positively correlated with MAPK signalling, ErbB signalling and glycolysis-related pathways but negatively correlated with the P53 signalling pathway (Fig. 7D). Our results indicated that high expression of ZDHHC9 had a negative effect on OS, relapse-free survival (RFS), disease-specific survival (DSS) and progression-free survival (PFS) in breast cancer patients in a multi-centre retrospective cohort (Fig. 7E). Additionally, the high expression of ZDHHC9 was accompanied by TP53 mutations and subsequent mutations in genes such as CDH1 and MAP3K1 (Fig. 7F).

### Expression of ZDHHC9 in TNBC tissues and cell lines and its clinical significance

**Fig. 8:**
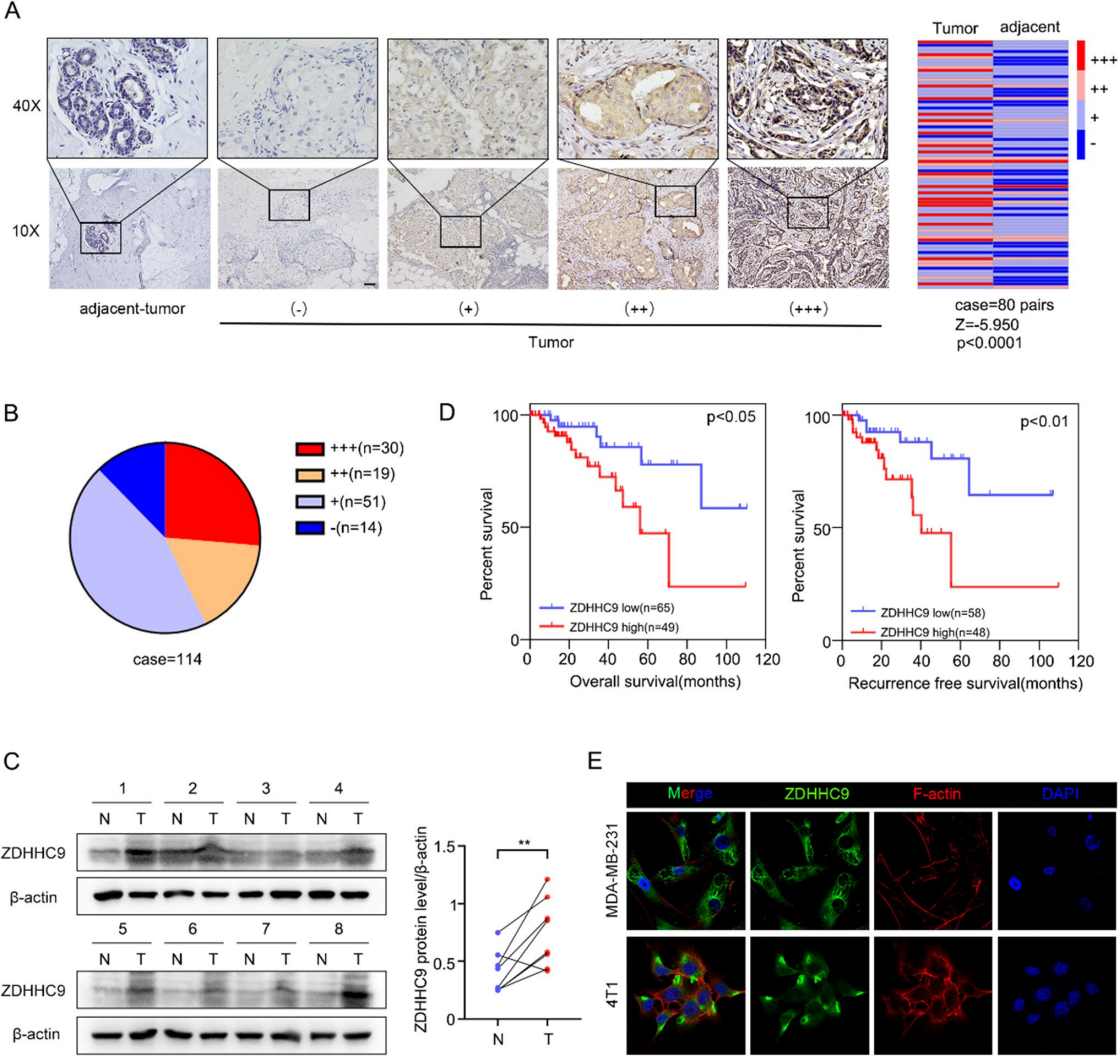
Expression of ZDHHC9 in TNBC tissues and cell lines and its clinical significance. **A** Representative images of staining for ZDHHC9 in TNBC and normal tissues: no expression (−), weak expression ( +), moderate expression (+ +) and strong expression (+ + +). The differential expression of ZDHHC9 in 80 pairs of tumor and normal tissues is shown in the heatmap and statistically analysed using the Wilcoxon signed-rank test. **B** Distribution of immunohistochemical staining intensity of ZDHHC9 in 114 TNBC tissues. **C** Western blotting was performed to assess the expression of ZDHHC9 in 8 pairs of TNBC tissues (T) and paraneoplastic normal tissues (N). ZDHHC9 protein was quantified in TBNC tissues and paired paraneoplastic normal tissues in greyscale, and ZDHHC9 expression was normalised to β-actin expression. **D** Kaplan–Meier analysis was performed for assessing the OS and RFS of patients with TNBC with high and low ZDHHC9 expression. Moderate and strong expression were defined as high expression, and others were defined as low expression. **E** Representative images of immunofluorescence staining for ZDHHC9 (green) and F-actin (red) in the human TNBC cell line MDA-MB-231 and the murine TNBC cell line 4T1

To assess the expression pattern of ZDHHC9 in TNBC, we performed IHC staining using 80 pairs of TNBC and paraneoplastic tissue specimens. The results revealed that ZDHHC9 expression was higher in TNBC tissues than in paraneoplastic tissues (Fig. 8A). Additionally, positive expression of ZDHHC9 was predominant in TNBC tissues (Fig. 8B), and western blotting also showed that ZDHHC9 was abnormally overexpressed in TNBC tissues (Fig. 8C). Furthermore, the expression of ZDHHC9 was significantly correlated with the pT stage, pN stage, and pTNM stage in patients with TNBC (Table 1). The clinical significance of ZDHHC9 was examined by analysing the OS and RFS of patients with TNBC. High expression of ZDHHC9 was found to have a negative impact on both OS and RFS (Fig. 8D). ZDHHC9 expression was detected in the cytoplasm of both human TNBC MDA-MB-231 cells and murine TNBC 4T1 cells (Fig. 8E), which is consistent with the results of public data. Altogether, these results suggest that ZDHHC9 is overexpressed in TNBC and its overexpression is associated with a poor prognosis.

**Table 1:** Association between ZDHHC9 expression and clinicopathological features of patients with TNBC

	Total	ZDHHC9 expression			
		Low	High	χ^2^	*P* value
Age(year)					
< 55	79	37	24	0.709	0.4
≥ 55	75	28	25		
pT staging					
T1-T2	89	57	32	8.178	0.004*
T3-T4	25	8	17		
pN staging					
N0	83	53	30	5.823	0.016*
N1-N3	31	12	19		
pTNM staging					
I–II	93	58	35	5.892	0.015*
III	21	7	14		

^*^p < 0.05 (Chi-Square Test)

### ZDHHC9 expression in tumors decreased the abundance of CD8+T cells and NK cells and increased the abundance of PD1+CD8+T cells and Tim3+CD8+T cells

To investigate the role of ZDHHC9 in immunity in TNBC, it was knocked down in 4T1 cells via shRNA transfection. The transfection efficiency of shZDHHC9 1# was robustly validated through western blotting, qRT-PCR and IF assays (Supplementary Fig. 4A–C).

**Fig. 9:**
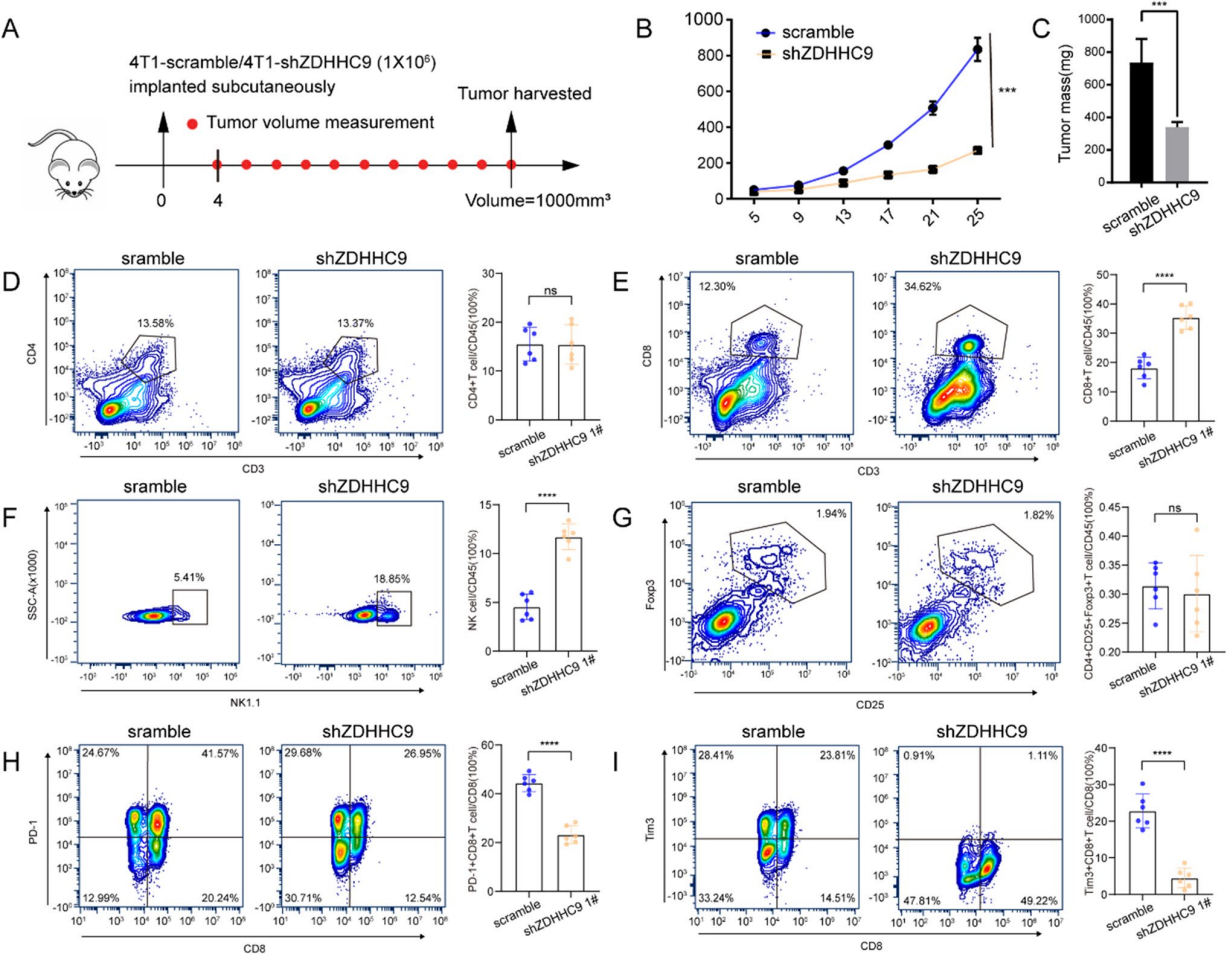
ZDHHC9 expression in tumors decreased the abundance of CD8 + T cells and NK cells and increased the abundance of Treg cells, PD1 + CD8 + T cells and Tim3 + CD8 + T cells. **A** Immunocompetent C57BL/6 mice were subcutaneously injected with 4T1-scramble or 4T1-shZDHHC9 cells. Tumor size was measured from day 4 after inoculation until the mice were sacrificed when the tumor volume reached 1,000 mm^3^, and tumor tissues were subsequently harvested. **B**,** C** Statistical analysis of tumor growth curves and tumor tissue weight in the 4T1-scramble and 4T1-shZDHHC9 groups. **D**–**I** Representative contour plots (left) and statistical analysis (right) of the abundance of tumor-infiltrating CD4 cells, CD8 cells, NK cells, Treg cells, PD1 + CD8 + T cells, and Tim3 + CD8 + T cells in the 4T1-scramble and 4T1-shZDHHC9 groups. Data are expressed as the mean ± SD. An unpaired t-test was used for statistical analysis (***p < 0.001; ****p < 0.0001; ns, not significant)

Mouse 4T1-scramble and 4T1-shZDHHC9 cells were subcutaneously injected into immunocompetent C57BL/6 mice, and subcutaneous tumors were harvested after the tumor volume reached approximately 1000 mm^3^ (Fig. 9A). The growth of subcutaneous tumors was significantly reduced in 4T1 cells with ZDHHC9 knockdown (Fig. 9B, C).

Furthermore, immune cell infiltration within the tumor tissue was analysed via flow cytometry. Specifically, the relative frequencies of CD4 + T cells, CD8 + T cells, NK cells and Treg cells in CD45 + leukocytes were evaluated. The infiltration levels of CD8 + T cells (scramble, 18.15%; shZDHHC9, 35.38%; p < 0.0001) and NK cells (scramble, 4.533%; shZDHHC9, 11.71%; p < 0.0001) were significantly higher in 4T1-shZDHHC9 tumor tissues, whereas those of CD4 + T cells and Treg cells were similar between 4T1-scramble and 4T1-shZDHHC9 tumor tissues (Fig. 9D–G). Additionally, the infiltration levels of PD-1 + CD8 + T cells (scramble, 44.4%; shZDHHC9, 23.23%; p < 0.0001) and Tim3 + CD8 + T cells (scramble, 22.81%; shZDHHC9, 4.508%; p < 0.0001) were significantly lower in the 4T1-shZDHHC9 group than in the 4T1-scramble (control) group (Fig. 9H, I).

IF analysis was performed (CD8/NCR1, ZDHHC9 and DAPI) in TNBC tissue specimens (Supplementary Fig. 5A–B). The results revealed that the proportion of CD8 + T cells and NK cells was significantly lower in the high-ZDHHC9-expression group than in the low-ZDHHC9-expression group, which is consistent with the results of flow cytometry. Altogether, these results suggest that ZDHHC9 plays a negative regulatory role in the infiltration of CD8 + T cells and NK cells within tumor tissues.

Notably, the expression level of ZDHHC9 played a significant role not only in shaping the immune microenvironment of TNBC tumors but also in restraining the proliferation, invasion, and metastatic potential of TNBC cells upon downregulation of ZDHHC9 expression (Supplementary Fig. 6A–D).

## Discussion

Given that TNBC has limited treatment options and a poor prognosis [3], it is necessary to identify new molecular targets for the development of effective therapies.

This study revealed four genes that were highly expressed in TNBC and were associated with immunosuppression and resistance to ICB therapy. These genes include TBC1D24, TRIM67, QRSL1 and ZDHHC9. ZDHHC9, which has been associated with colorectal cancer and glioblastoma [15, 21], was upregulated in breast cancer tissues, especially TNBC tissues, compared with other molecular subtypes of breast cancer. To predict the cancer-promoting characteristics and prognosis of TNBC using the combination of the four genes, we calculated risk coefficients for co-expression integration and constructed a risk model.

WGCNA of TNBC samples with clinical features revealed that ZDHHC9 was a critical risk factor for TNBC. Enrichment analyses revealed that critical pathways such as TGFB1, Hippo and MAPK signalling pathways and focal adhesion-related pathways were enriched in the cell cluster with high expression of ZDHHC9. In public datasets, patients with high ZDHHC9 expression had an increased proportion of Ki-67 + breast cancer cells and tended to have basal-like breast cancer. Basal-like breast cancer, a subtype of breast cancer identified in cDNA microarray studies, has a basal-like phenotype both morphologically and immunophenotypically. Approximately 90% of basal-like breast cancer cases are TNBC [22]. Basal-like breast cancer cannot be treated with endocrine or targeted therapy owing to the lack of corresponding targets and is currently treated mainly with chemotherapy, which has poor treatment effects. Patients with basal-like breast cancer have high rates of early local recurrence and distant metastasis and low rates of DFS and OS [23]. Ki-67 is an indicator related to cell proliferation; its higher expression indicates faster tumor growth, poorer tissue differentiation and a worse survival prognosis [24, 25]. Previous studies have suggested that higher levels of Ki-67 in patients with early-stage breast cancer are associated with a worse prognosis and can accurately determine whether breast cancer has metastasised, thus providing a valuable reference for clinical diagnosis and treatment [26, 27]. In this study, IHC analysis and western blotting revealed that elevated expression of ZDHHC9 was associated with a poor prognosis in TNBC.

ZDHHCs are known to be expressed in T cells, where they mediate palmitoylation of T-cell proteins, thus playing an essential role in the activation and differentiation of T cells [28]. Recent studies have highlighted the role of ZDHHC3-mediated palmitoylation of PD-L1 in colorectal cancer, which blocks PD-L1 degradation by lysosomes and suppresses antitumor immunity [29]. In addition, ZDHHC9-mediated palmitoylation maintains the stability and cell surface distribution of PD-L1 protein, enabling immune escape of breast cancer cells [16]. In this study, we found that ZDHHC9 was associated with the immunosuppressive microenvironment and immunotherapeutic resistance in TNBC, which affected the infiltration of multiple immune cells, including NK and CD8+T cells.

Altogether, this study suggests that ZDHHC9 is a valuable marker for guiding the classification, diagnosis and prognosis of TNBC and developing specific targeted therapies. However, selective inhibitors of ZDHHCs are unavailable at present. The use of 2-bromopalmitate (2-BP), a broad-spectrum palmitoylation inhibitor, has been shown to sensitise patients with colorectal cancer to ICB therapy [30, 31]. Competitive inhibitors of PD-L1 palmitoylation, such as the CPP-S1 peptide, can reduce PD-L1 expression in tumor cells and enhance T-cell immunity against tumors [29]. Proteolysis-targeting chimera (PROTAC) is a valuable technique that enables targeted protein degradation through a two-component system comprising a ligand for the target protein and another for a covalently linked E3 ubiquitin ligase. The target protein ligand within the PROTAC molecule binds to its designated protein and facilitates its degradation via ubiquitination [32]. In our forthcoming research, our objective is to utilize the PROTAC technology in the development of a specific ZDHHC9 inhibitor for potential clinical translation.

Despite the numerous novel findings of this study, some limitations should be addressed in future studies. In particular, the mechanisms underlying the effects of ZDHHC9 on immune cell infiltration in breast cancer warrant further investigation and validation. Additionally, in addition to its impact on the immune function of TNBC cells, ZDHHC9 expression also plays a crucial role in enhancing their proliferative potential and promoting invasive metastasis. However, the precise underlying mechanisms responsible for these effects require further investigation. Although this study focused on screening potential therapeutic targets through bioinformatic analysis and preliminary experimental validation, further research is required to overcome the abovementioned limitations.

### Supplementary Information

Additional file1 (DOC 4408 KB)

Additional file2 (XLSX 12 KB)

Additional file3 (XLSX 9 KB)

Additional file4 (XLSX 11 KB)

## Data Availability

The data supporting the findings of this study are available from the corresponding author upon reasonable request.
