# Supplementary material for: ZDHHC9: a promising therapeutic target for triple-negative breast cancer through immune modulation and immune checkpoint blockade resistance
Source: Discov Oncol. 2023 Oct 24;14:191. doi: 10.1007/s12672-023-00790-4 (PMC10597932; doi:10.1007/s12672-023-00790-4)
Supplement: Supplementary file 1 — Additional file1 (DOC 4408 KB) [file 12672_2023_790_MOESM1_ESM.doc]

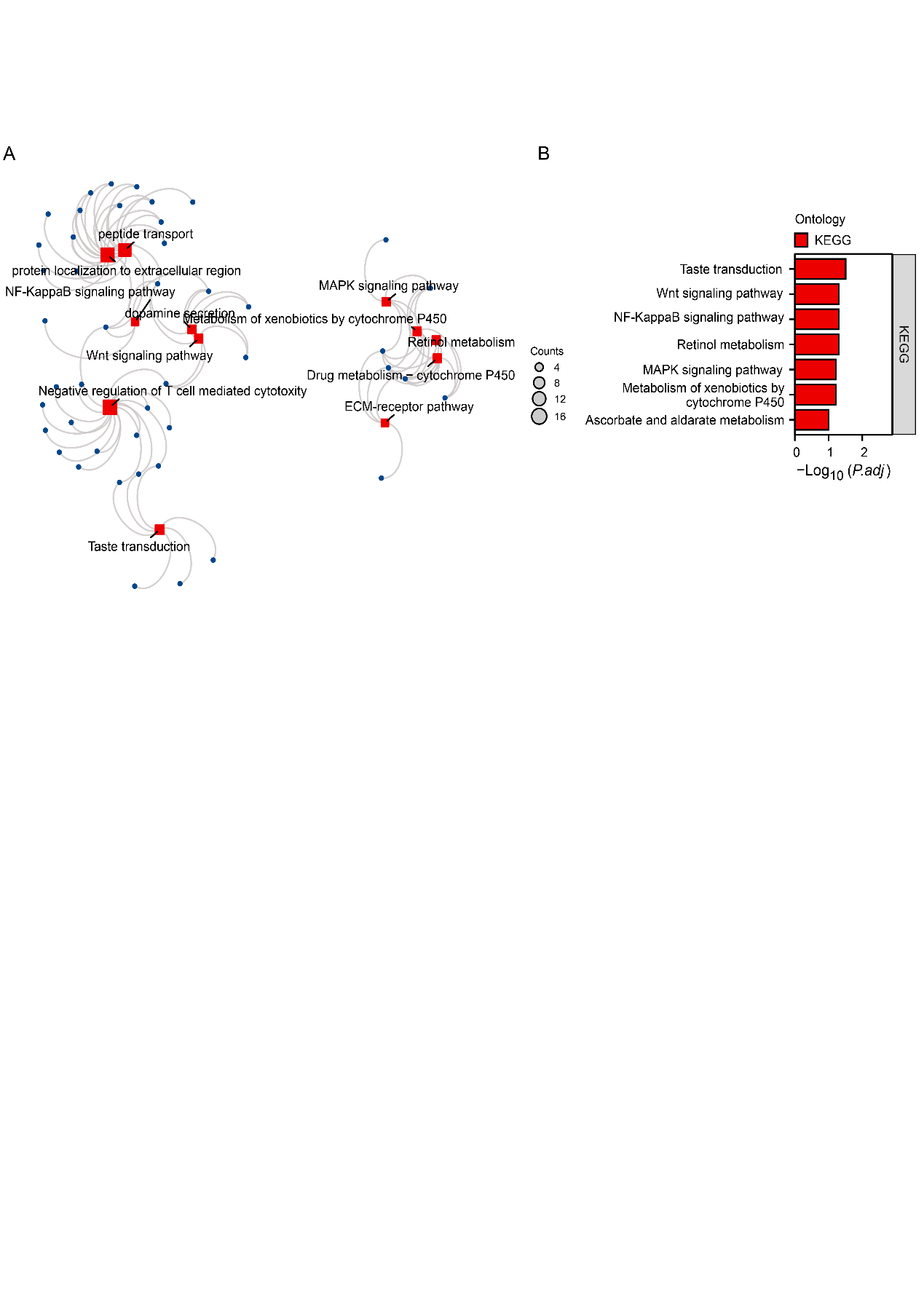


**Supplementary Fig.1 a-b** KEGG and GO pathway enrichment analyses of co-expressed genes. The genes are enlisted in Supplementary Table 1.


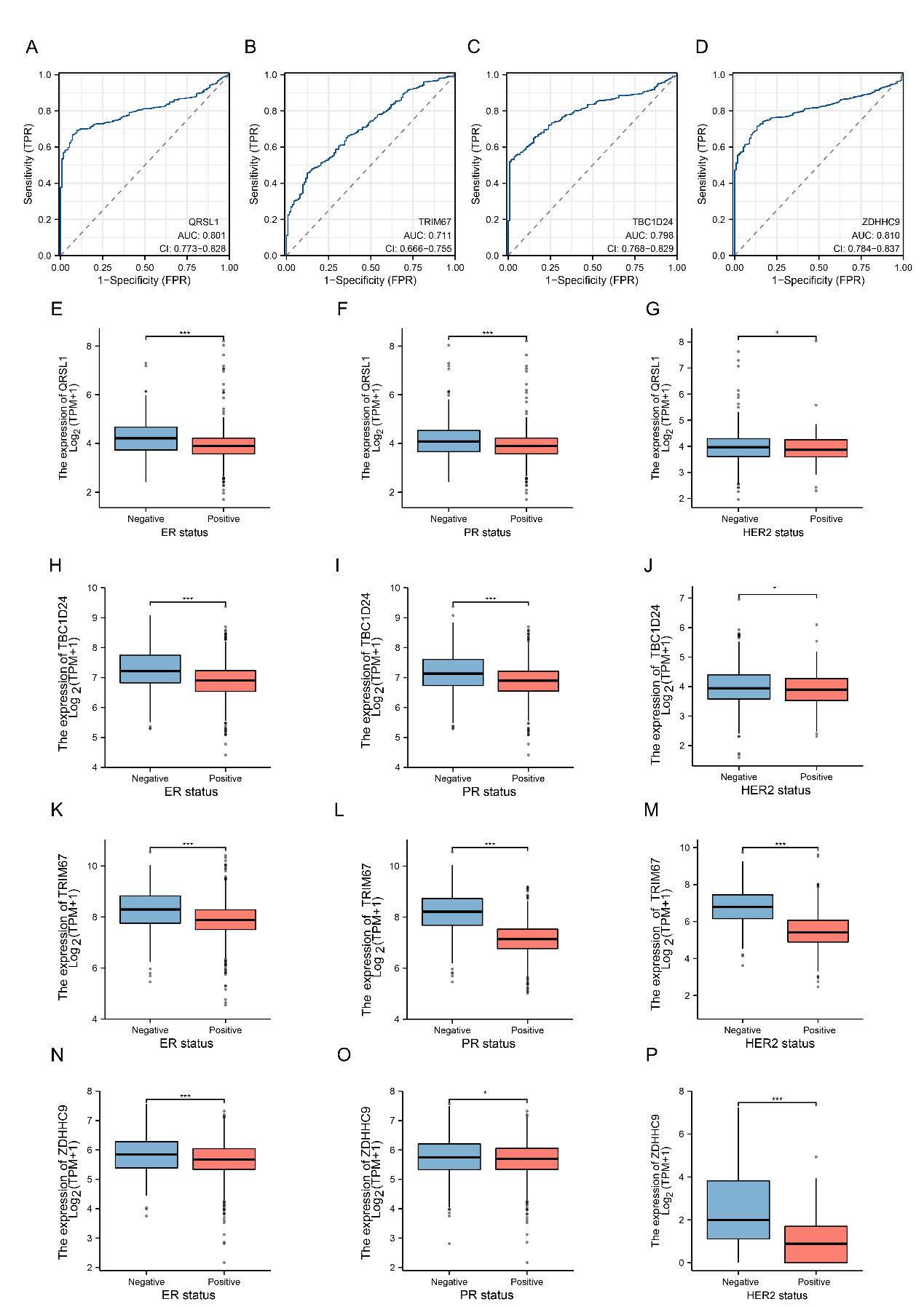


**Supplementary Fig.2 a-d** ROC curve analysis of QRSL1, TRIM67, TBC1D24, and ZDHHC9 for predicting the diagnosis of breast cancer. **e-p** Expression levels of the four genes in patients with ER-negative, PR-negative and HER-2-negative breast cancer.

**
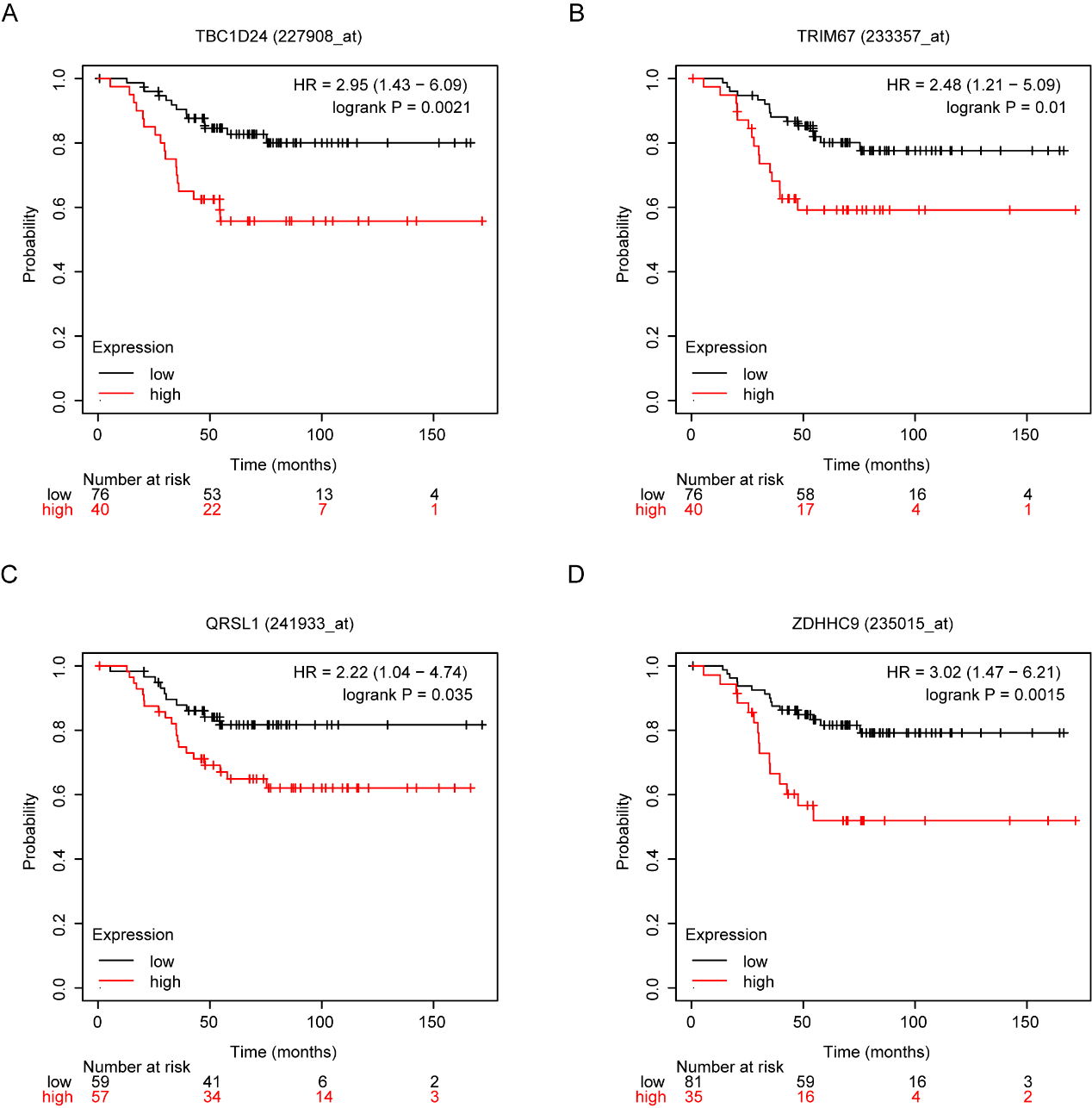
**

**Supplementary Fig.3** **a-d** K-M analysis for predicting the OS of patients with TNBC with different expression of the four genes.


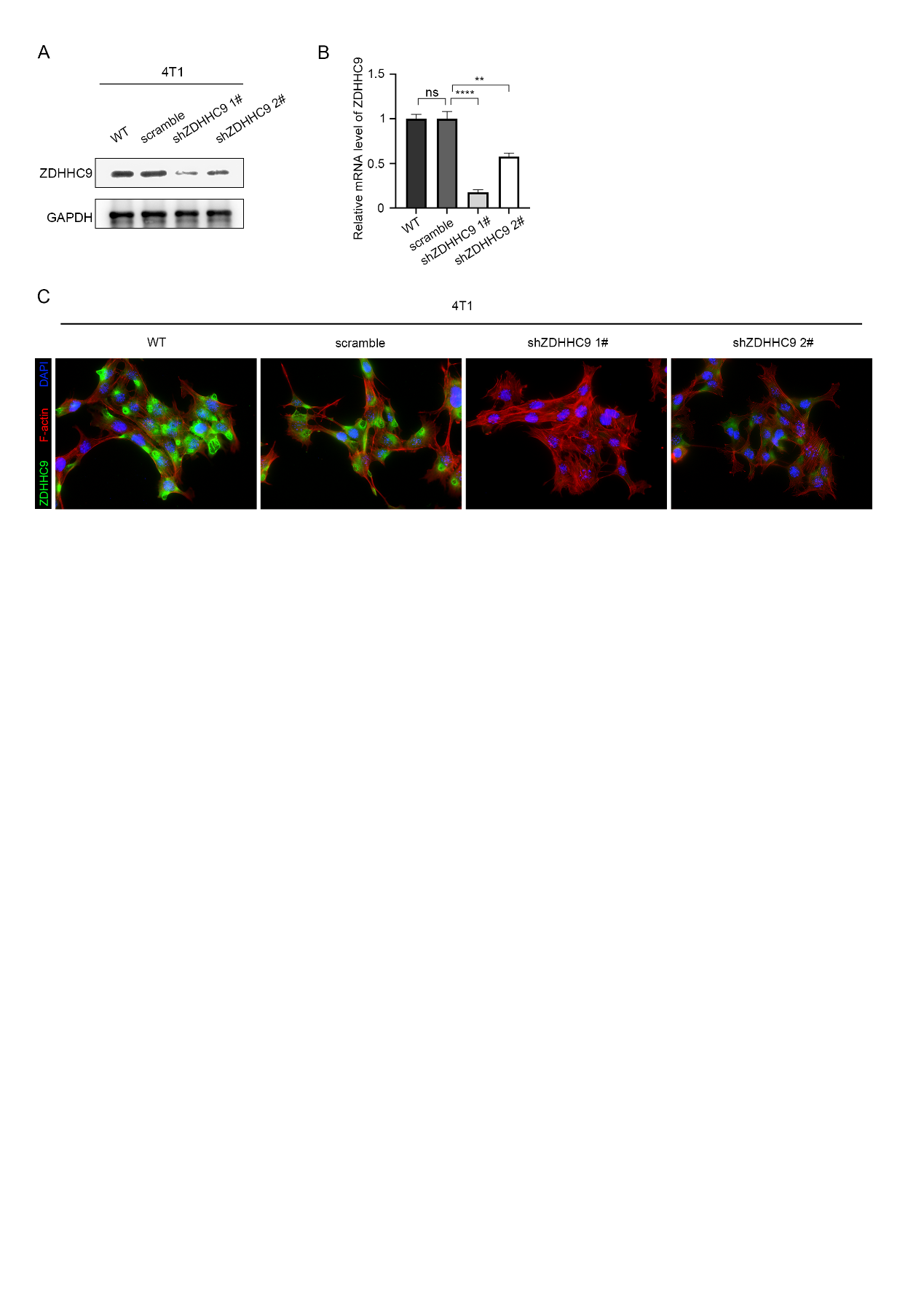


**Supplementary Fig.4 a-b** Western blotting and qRT-PCR were performed to detect the protein and mRNA expression of ZDHHC9 in normal (wild-type [WT]) 4T1 cells, 4T1 cells transfected with scramble shRNA, those transfected with shZDHHC9 (1#) and those transfected with shZDHHC9 (2#). Data are expressed as the mean± SEM from three independent experiments (**, p < 0.01; ****, p < 0.0001; ns, not significant; p > 0.05 [unpaired t-test]). **c** Immunofluorescence images of normal 4T1 cells and 4T1 cells transfected with scramble, shZDHHC9 (1#) and shZDHHC9 (2#). ZDHHC9, green; F-actin, red; DAPI, blue; magnification, 600×.


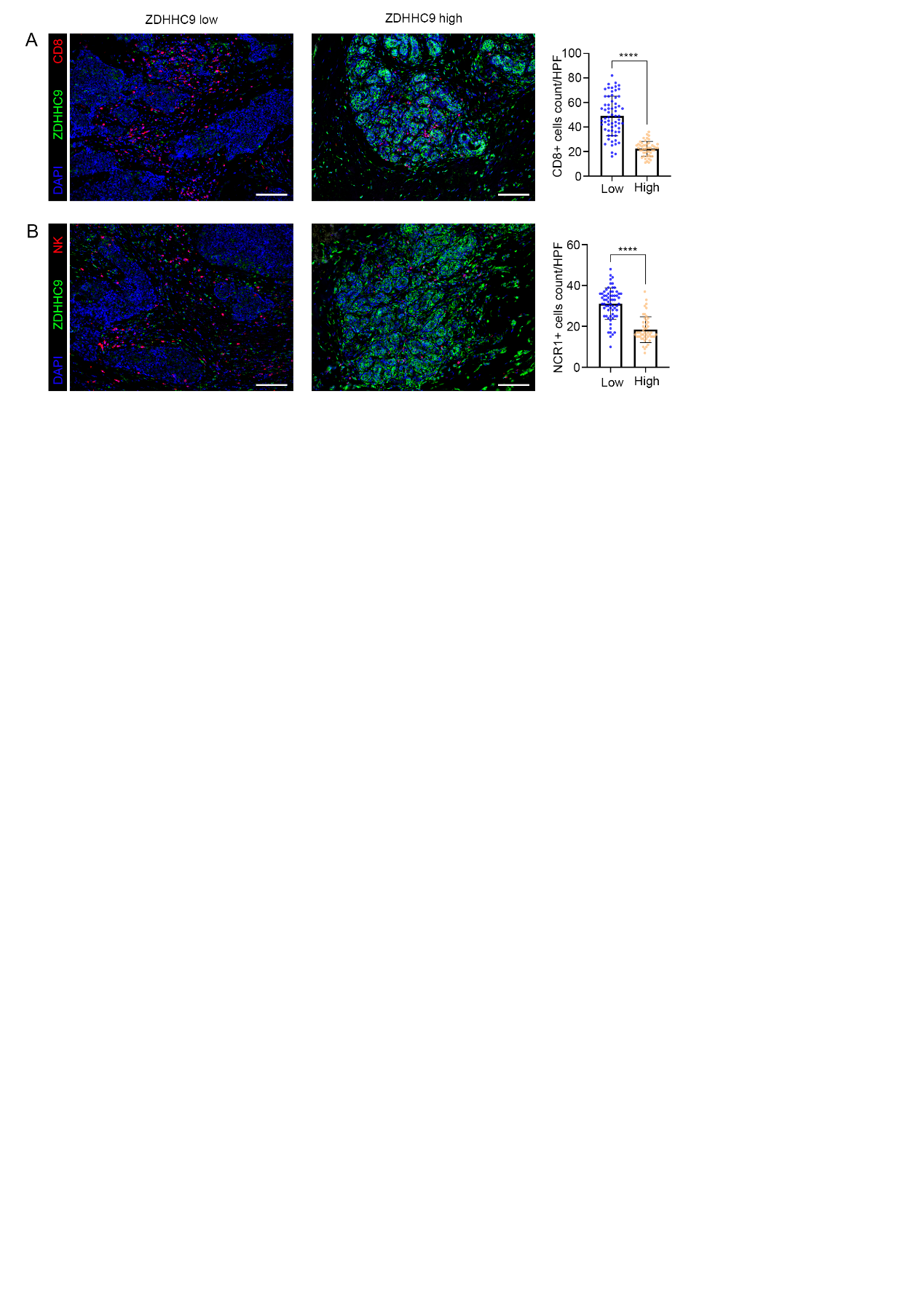


**Supplementary Fig.5 a-b** Representative images (left) of IF staining for ZDHHC9 expression and accumulation of CD8+ T cells, NCR1+ NK cells in TNBC tissues. An unpaired t-test was used for statistical analysis (right) (n = 70; ****, p < 0.0001; scale bar = 100 μm).


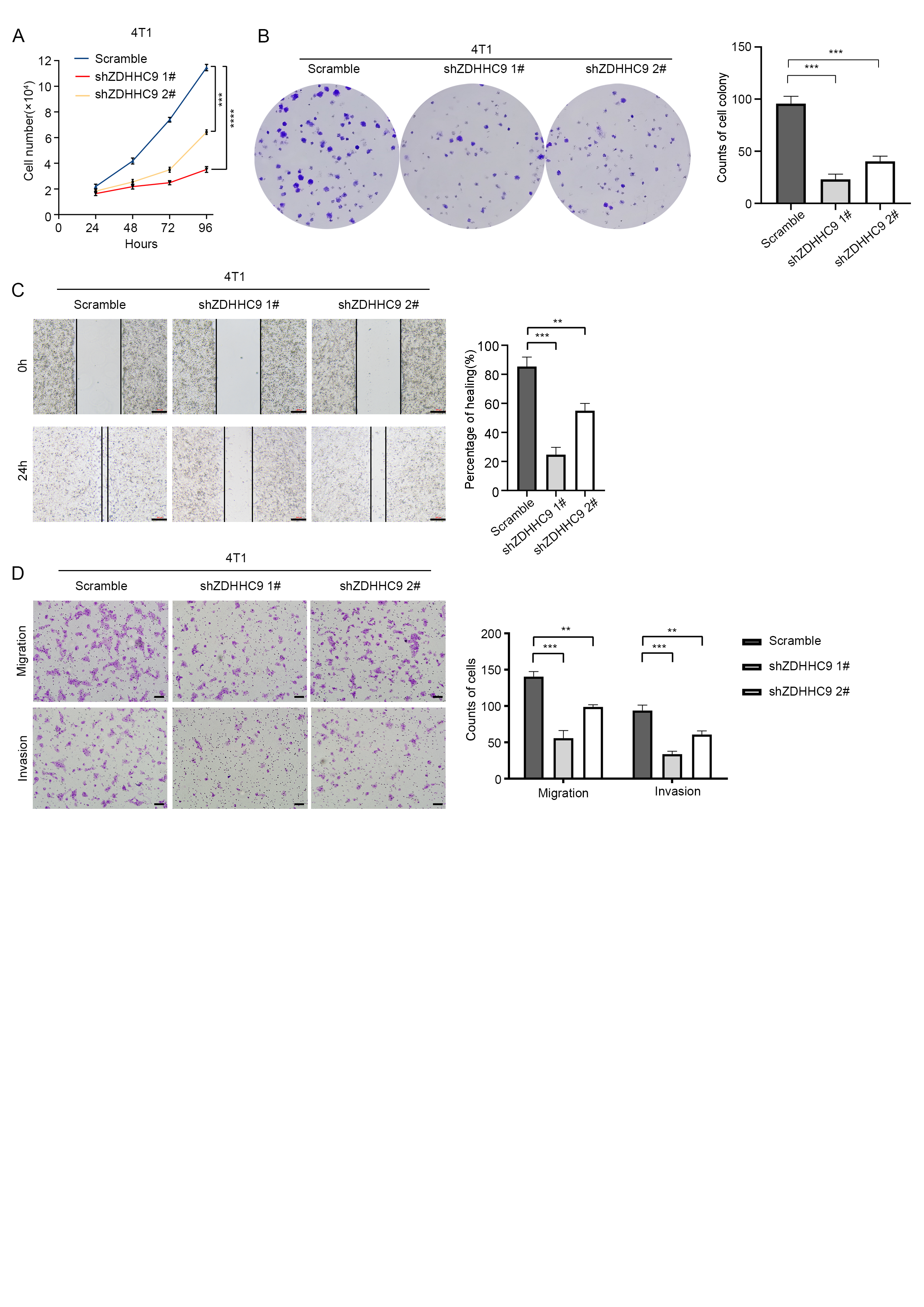


**Supplementary Fig.6** The Effect of ZDHHC9 Expression on Proliferation and Invasive Metastasis of Triple-Negative Breast Cancer (TNBC) Cells. **a** The proliferation of 4T1 cells transfected with scramble, shZDHHC9 (1#), and shZDHHC9 (2#) was assayed. The data are expressed as the mean ± SD from three independent experiments (***, p < 0.001; ****, p < 0.0001 [unpaired t-test]). **b** Clone formation of 4T1 cells transfected with scramble, shZDHHC9 (1#), and shZDHHC9 (2#) was examined. The data are expressed as the mean ± SD from three independent experiments (***, p < 0.001 [unpaired t-test]). **c** The motility of 4T1 cells transfected with scramble, shZDHHC9 (1#), and shZDHHC9 (2#) was examined using the wound healing assay. The percentage of wound healing was analyzed using an unpaired t-test. The data are presented as means ± SD, n = 3. **, p < 0.01; ***, p < 0.001. Scale bar, 100μm. **d** The migration and invasion abilities of 4T1 cells transfected with scramble, shZDHHC9 (1#), and shZDHHC9 (2#) were detected using transwell assays. The data are presented as means ± SD, n = 3. **, p < 0.01; ***, p < 0.001 in unpaired t-test. Scale bar, 100μm.
